# A drug repurposing screen reveals dopamine signaling as a critical pathway underlying potential therapeutics for the rare disease DPAGT1-CDG

**DOI:** 10.1371/journal.pgen.1011458

**Published:** 2024-10-28

**Authors:** Hans M. Dalton, Naomi J. Young, Alexys R. Berman, Heather D. Evans, Sydney J. Peterson, Kaylee A. Patterson, Clement Y. Chow

**Affiliations:** Department of Human Genetics, University of Utah School of Medicine, Salt Lake City, Utah, United States of America; RIKEN Advanced Science Institute, JAPAN

## Abstract

DPAGT1-CDG is a Congenital Disorder of Glycosylation (CDG) that lacks effective therapies. It is caused by mutations in the gene *DPAGT1* which encodes the first enzyme in N-linked glycosylation. We used a *Drosophila* rough eye model of DPAGT1-CDG with an improperly developed, small eye phenotype. We performed a drug repurposing screen on this model using 1,520 small molecules that are 98% FDA/EMA-approved to find drugs that improved its eye. We identified 42 candidate drugs that improved the DPAGT1-CDG model. Notably from this screen, we found that pharmacological and genetic inhibition of the dopamine D2 receptor partially rescued the DPAGT1-CDG model. Loss of both dopamine synthesis and recycling partially rescued the model, suggesting that dopaminergic flux and subsequent binding to D2 receptors is detrimental under *DPAGT1* deficiency. This links dopamine signaling to N-glycosylation and represents a new potential therapeutic target for treating DPAGT1-CDG. We also genetically validate other top drug categories including acetylcholine-related drugs, COX inhibitors, and an inhibitor of NKCC1. These drugs and subsequent analyses reveal novel biology in DPAGT1 mechanisms, and they may represent new therapeutic options for DPAGT1-CDG.

## Author summary

Some proteins require the attachment of sugars—like mannose and galactose—in order to perform their function. The process that attaches these sugars is called "glycosylation". Genetic mutations in glycosylation pathways cause Congenital Disorders of Glycosylation ("CDGs"). CDGs are devastating disorders causing a multitude of symptoms, including seizures and developmental delay, and they have few treatment options. We wanted to find new potential treatment options for the CDG called DPAGT1-CDG. We use a model of DPAGT1-CDG that has inhibited glycosylation resulting in impaired eye development. We used a "drug repurposing screen", where we screened 1,520 drugs that are 99% FDA- or EMA-approved, to find drugs that improved its eye. We found 42 drug hits, including classes like dopamine receptor inhibitors, NSAIDs, and antihistamines. We found that the dopamine receptor inhibitors caused the strongest improvement. In addition, genetically impairing multiple parts of the dopamine pathway (e.g. how dopamine is made or recycled) also improved the model. This suggests that dopamine signaling is important in DPAGT1-CDG, and it could represent a new therapeutic option for patients. Overall, we present validation of several of our drugs, and these represent potential new treatments while also improving our understanding of glycosylation.

## Introduction

Nearly 10% of the US population has a rare disease, yet over 95% lack effective therapeutics [1,2]. One solution to find new therapeutics is drug repurposing which use drugs that are already approved or under investigation [3]. When successful, drug repurposing screens provide immediate potential therapeutics that have already passed the rigor of human safety trials and may have a faster route of clinical approval [3]. Alternatively, they can potentially be used in an off-label capacity at a clinician’s discretion [3–5]. Even if a repurposed screen does not immediately result in a new therapy, it can still identify new biological interactions that can increase our understanding of a disorder.

DPAGT1-CDG is a rare disease that results from autosomal recessive loss-of-function mutations in the gene encoding the enzyme DPAGT1. It is one of nearly 200 Congenital Disorders of Glycosylation (CDGs) [6–9]. Glycosylation includes multiple pathways where sugars are co- or post-translationally added to proteins, lipids, or RNAs [10,11]. For proteins, it is critical for their proper localization, folding, and function [10]. DPAGT1 is part of the N-glycosylation pathway where these sugars are added to specific asparagine (N) residues, typically part of the canonical consensus sequence N-X-S/T where X is a non-proline amino acid [12]. DPAGT1 synthesizes dolichol-PP-GlcNAc which is the first step in N-glycosylation [6].

DPAGT1-CDG causes developmental delay, muscle weakness, and seizures, among other symptoms [6,13,14]. Less severe mutations in *DPAGT1* can cause a form of congenital myasthenia syndrome (CMS) called DPAGT1-CMS [15]. Unlike DPAGT1-CDG, the CMS form has a known disease mechanism caused by hypoglycosylation of acetylcholine and calcium receptors [15,16]. Acetylcholinesterase inhibitors that increase acetylcholine levels can alleviate muscle weakness symptoms in both DPAGT1-CDG and -CMS patients [17–19]. For treating seizures, antiepileptic drugs have also been prescribed for DPAGT1-CDG and other CDGs [20]. However, both of these treatments are palliative, and there remains a great need for better therapeutics for the multisystemic symptoms in DPAGT1-CDG.

*Drosophila* share ~75% of human disease-causing genes [21] and have been regularly used in drug screens [22–24]. Drug repurposing screens in *Drosophila* have had previous success in identifying new potential therapies for CDGs. For example, our laboratory identified GSK3β inhibitors as a potential treatment for NGLY1 deficiency [25]. In addition, a drug repurposing screen in *C*. *elegans* identified the aldose reductase inhibitor epalrestat for treating PMM2-CDG [26,27]. Thus, a drug repurposing screen in *Drosophila* for DPAGT1-CDG could help find new potential therapeutics for this disorder.

In this study, we performed a drug repurposing screen on a *Drosophila* model of DPAGT1-CDG to identify new therapeutics and biological interactions with DPAGT1. We identified 42 compounds that suppress the DPAGT1-CDG model and genetically confirmed many of the drug interactions. These 42 compounds include acetylcholine-related drugs, dopaminergic antagonists, and cyclooxygenase inhibitors, among others. Importantly, we find that both pharmacologic and genetic inhibition of the D2 receptor can partially rescue our *DPAGT1* model. In addition, impairing dopamine (DA) synthesis or recycling can also partially rescue the model. Our data suggest that manipulating DA signaling is a promising therapy for DPAGT1-CDG and implicates a larger role of DA signaling on N-glycosylation. The compounds and pathways identified in this screen are potential new therapeutic and genetic interactions with DPAGT1 that may represent new treatment options for DPAGT1-CDG.

## Results

### Identifying suppressors of a DPAGT1-CDG model in a primary drug repurposing screen

**Fig 1:**
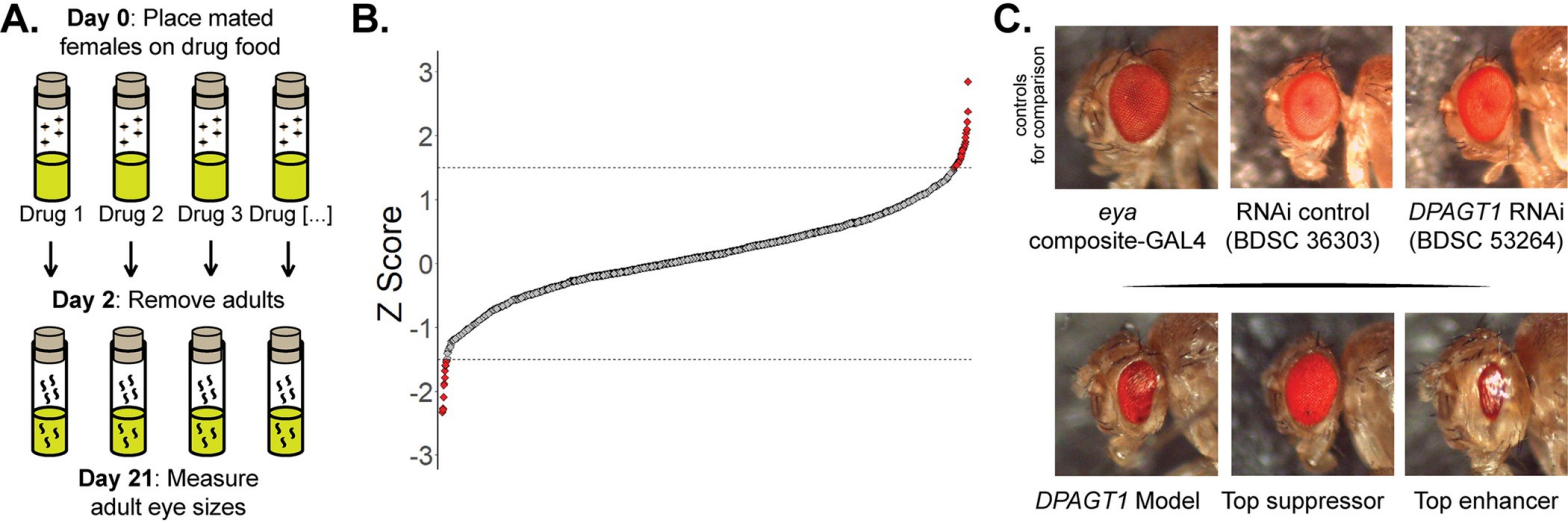
Summary of the drug repurposing screen. **(A)** Screening method for the drug repurposing screen. **(B)** Z-score plot of all repurposed drugs tested. Each point is the Z-score of a drug compared to the average of DMSO-treated control flies. Dotted lines indicate our Z-score threshold of 1.5 or -1.5. We found 42 and 16 drugs with a Z-score of ≥1.5 or ≤-1.5, respectively. **(C)** Representative images showing male *Drosophila* stocks of the *eya* composite-GAL4 driver (control, without RNAi), the RNAi background control to the *DPAGT1* model (attP2), the *DPAGT1* RNAi alone (control, without a GAL4 driver), the *DPAGT1* model, and the effects of the top suppressor (Z-score = 2.85) and top enhancer (Z-score = -2.92). See S1 and S2 Tables for a complete list of compounds and Z-scores, and S2 Fig for representative female images.

To model DPAGT1-CDG, we used an eye-based *Drosophila* model that we previously established [28]. The *Drosophila* eye is regularly used to study and model biological processes, including development [29–32]. In our model, *DPAGT1* expression is reduced in the eye by RNA interference (RNAi, UAS-GAL4 system [33], *eya* composite-GAL4 driver [34]). Analysis of whole fly heads of the *DPAGT1* model resulted in a ~65% knockdown of *DPAGT1* expression (S1A Fig). The compound eye makes up a large portion of the *Drosophila* head, but this knockdown is likely even lower because our RNAi is eye-specific. *In vitro* analysis of DPAGT1-CDG patient proteins found they had 10–50% protein activity [35]. Thus, having at most ~35% of normal *DPAGT1* expression is a reasonable amount of knockdown to model *DPAGT1* deficiency. This knockdown results in a small, improperly developed, rough eye phenotype (hereafter referred to as “*DPAGT1* model”) [28] (Fig 1). To validate this small eye phenotype, we also created a second model using a different RNAi line (BDSC 51869) driven by *eya* composite-GAL4. This model has a small, rough eye phenotype comparable to our original model (S1B Fig).

The *DPAGT1* model can be chemically or genetically manipulated to make the eye larger (suppress the phenotype) or smaller (enhance the phenotype) [28]. To find potential therapeutics, we used the Prestwick Chemical Library, a collection of 1,520 compounds that are 98% FDA- or EMA-approved. We raised *DPAGT1* model flies on food containing each drug and measured the eye size of resulting progeny (Fig 1A). We used a 5 μM drug concentration—a standard dosage for flies that does not cause toxicity [25]. We scored 8,076 flies in total in the screen (4.4 average flies per treatment), and we calculated the Z-score of each compound by comparing their eye sizes to DMSO-treated *DPAGT1* model control flies. We identified 42 compounds that partially suppressed the eye phenotype of the *DPAGT1* model ("suppressors") (Fig 1B and 1C, S1 and S2 Tables) and 16 compounds that enhanced the eye phenotype ("enhancers") (Fig 1B and 1C, S1 and S2 Tables). 16 compounds resulted in no flies (S1 Table). Our positive hit rate of 2.8% falls in line with other compound screens performed in *Drosophila* [23,25,36,37].

We focused our validation efforts on suppressors that had ≥3 drugs in a particular drug class, had the strongest suppressive effect on eye size (Z-score ≥2), or had high potential for patient use (e.g. available over-the-counter). Drug classes with the highest representation were acetylcholine-related drugs, dopamine (DA) receptor antagonists, COX inhibitors, and antioxidants (three drugs each, S2 Table). In total, we tested 20/42 (48%) of the suppressors in validation experiments.

### Impairing acetylcholine breakdown improves the DPAGT1 model

**Fig 2:**
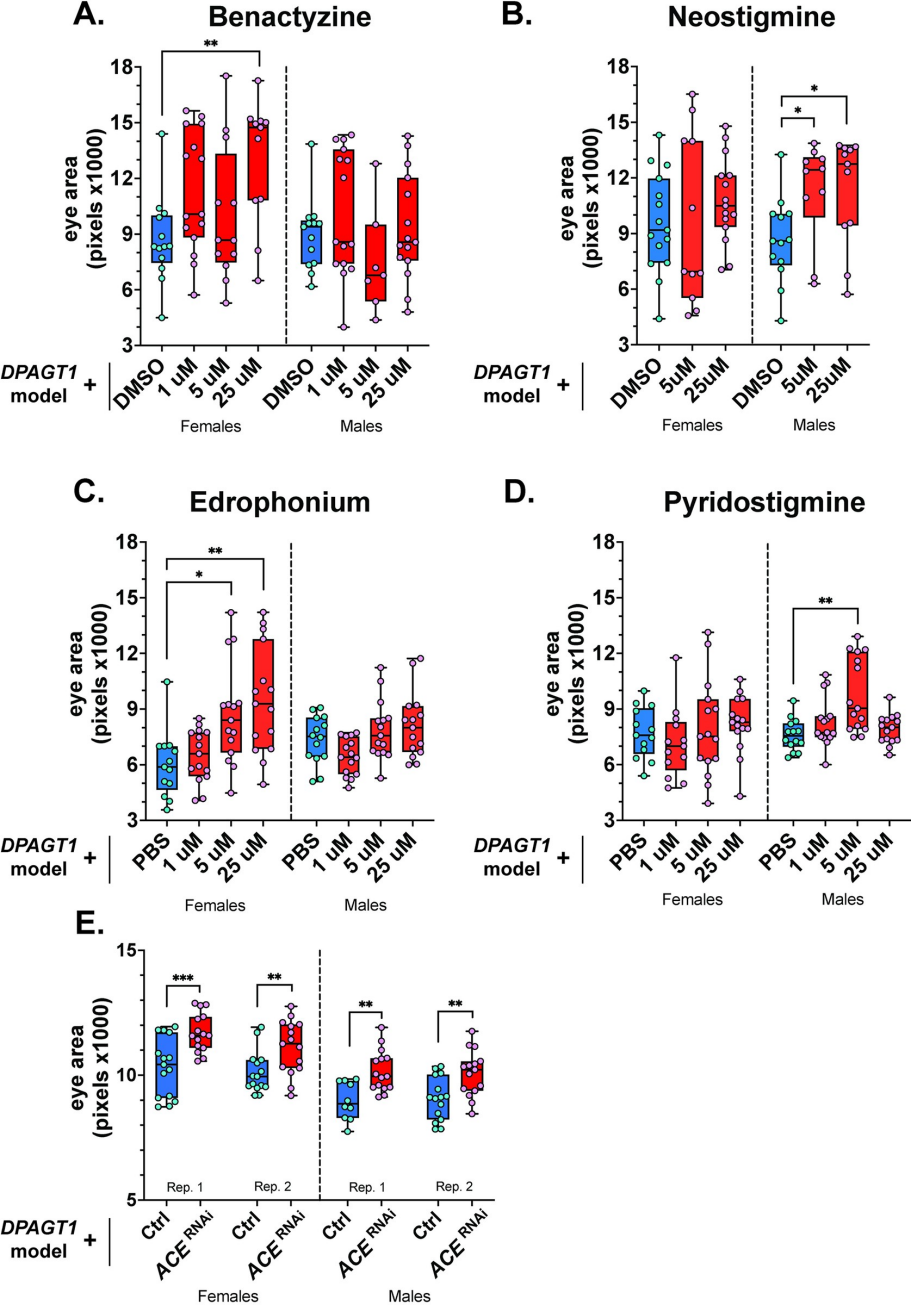
Pharmacological and genetic inhibition of acetylcholinesterase improved the *DPAGT1* model. **(A-D)** Multiple ACh-related drugs can partially rescue the *DPAGT1* model. For Neostigmine, we assayed 1 μM separately, but it was not significant. (**E)** RNAi knockdown of *ACE* also partially rescues the *DPAGT1* model (VDRC 105432KK). See S2 and S3 Tables for more details. * p<0.05, ** p<0.01, *** p<0.001 (Student’s t-test or One-way ANOVA with multiple comparison correction).

Mutations in *DPAGT1* can cause muscle weakness because acetylcholine receptors (AChRs) become hypoglycosylated [15,16]. This muscle weakness can be treated by cholinesterase inhibitors which increase available acetylcholine [15,16,38]. Our unbiased screen identified three AChR-related drugs. This included methacholine, an analog of acetylcholine and a muscarinic receptor agonist [39], as well as two drugs expected to increase acetylcholine levels: benactyzine, a cholinesterase inhibitor/anticholinergic [40], and neostigmine, a cholinesterase inhibitor [41]. To validate drug hits from the screen, we primarily used drugs purchased from a second source to reduce potential quality control errors (this is true of all further validation) (S2 Table). In dose-response analyses, benactyzine and neostigmine both increased the eye size in the *DPAGT1* model (Fig 2A and 2B). Benactyzine significantly increased female eye size at 25 μM and generally increased the upper distribution of eye sizes at most doses tested in both sexes (Fig 2A). Neostigmine significantly increased eye size in males at 5 and 25 μM (Fig 2B) and had a trending (+12%), but not statistically significant, increase in median size in females at 25 μM. As acetylcholinesterase inhibitors are already used in patients, these two drugs represent a strong validation of the screen to find patient-relevant hits.

We next assayed four acetylcholine-related drugs already used by DPAGT1-CDG or -CMS patients for their effect on the *DPAGT1* model. This included pyridostigmine (used in DPAGT1-CMS [17,19] and DPAGT1-CDG patients [18]), amifampridine, salbutamol, and edrophonium (all used in DPAGT1-CMS patients, though edrophonium is more diagnostic [17,42]). Treatment with edrophonium strongly improved female *DPAGT1* model flies in a dose-dependent manner (Fig 2C). Similar to its analog neostigmine (Fig 2B), treatment with pyridostigmine caused a significant increase in eye size in males at 5 μM (Fig 2D) and a small, but not significant increase in females at 25 μM (+6%). Overall, acetylcholine-affecting drugs identified in the screen and currently used by patients improved the *DPAGT1* model.

The rescuing drugs neostigmine, pyridostigmine, and edrophonium all inhibit the enzyme acetylcholinesterase (AChE) which breaks down acetylcholine [16]. We tested whether genetic inhibition of the AChE-encoding gene *ACE* (human: *ACHE*) could partially rescue the model as well. We used two RNAi lines against *ACE* (via the GAL4/UAS system [33]) to mimic pharmacological inhibition. One previously studied [43] RNAi line improved the *DPAGT1* model in both sexes (Fig 2E), mimicking the drug inhibition of *ACE*. The second RNAi line was capable of improving females (S3 Table). *ACE* knockdown on its own caused no effect in males, and only a 4–5% increase in females (see all *eya* composite-GAL4 control data in S3 Table). These pharmacological and genetic data indicate that inhibition of AChE, and the ostensible increase in acetylcholine, can partially rescue the eye development defect of the *DPAGT1* model. This validates the screen, supports continued usage of AChE inhibitors in patients, and might also indicate a role for AChE inhibitors outside of their effect on muscle weakness.

### Antagonizing D2 receptor signaling significantly improves the DPAGT1 model

**Fig 3:**
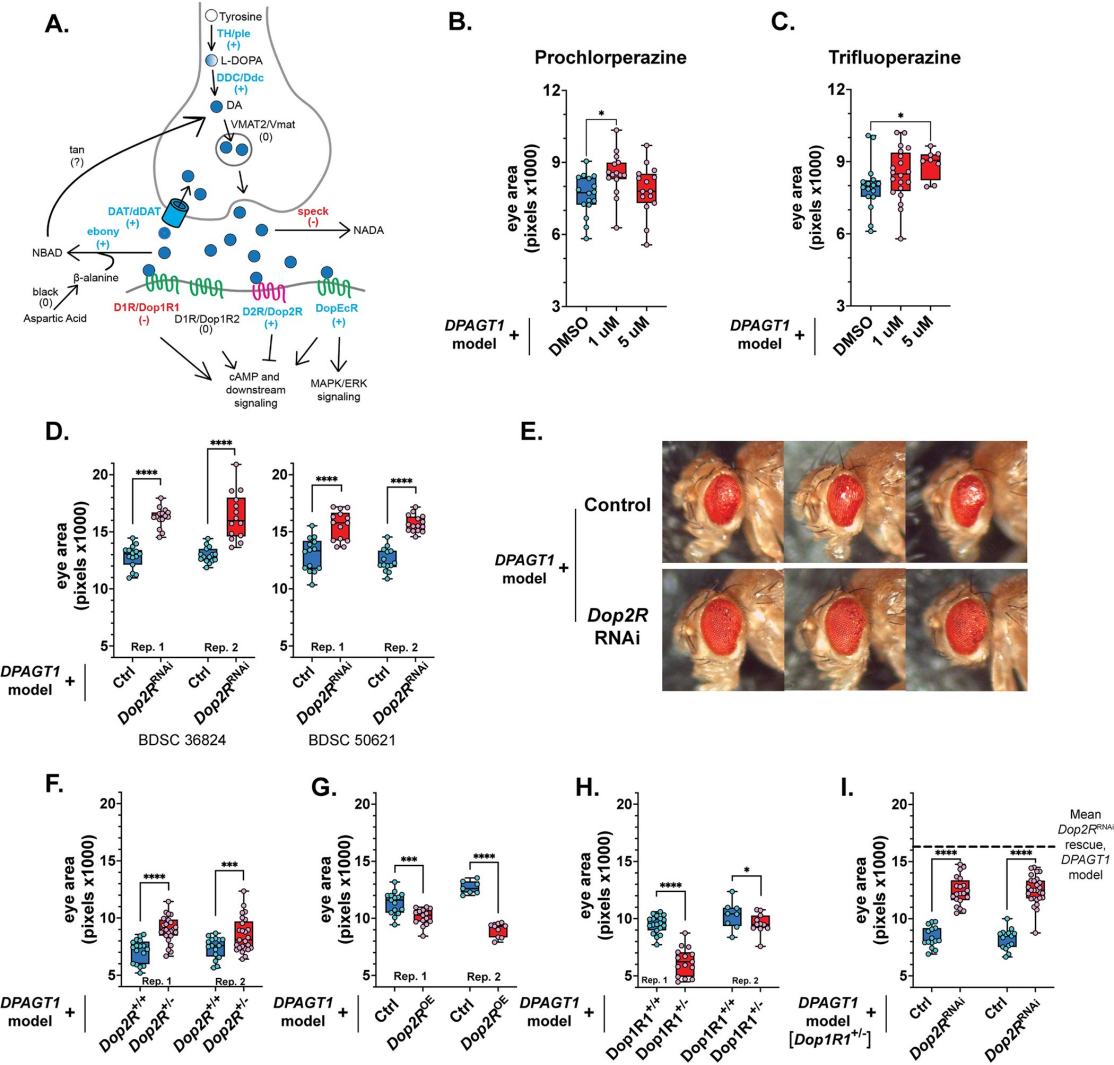
Inhibiting the dopamine 2 receptor improved the *DPAGT1* model. (**A)** A schematic of DA synthesis, recycling, metabolism, and signaling in *Drosophila*. The effect of each RNAi or mutation on the *DPAGT1* model is indicated. Genes in blue with the "+" symbol improved the *DPAGT1* model when knocked down, while those in red with the "-" symbol made it worse. Genes in black with the "0" symbol had no effect or could not be distinguished from the knockdown effect on its own. **(B-C)** The D2 antagonists prochlorperazine and trifluoperazine improved eye size in the *DPAGT1* model. For technical reasons, we assayed 25 μM separately, but neither drug was significant. **(D)** RNAi knockdown of the *Dop2R* gene (BDSC 36824) improved the *DPAGT1* model. **(E)** Representative images comparing the *DPAGT1* model crossed with the control RNAi background (attP2, BDSC 36303) or a *Dop2R* RNAi line (BDSC 36824). **(F)** *DPAGT1* model flies carrying a heterozygous null of *Dop2R* had increased eye size (BDSC 84720). **(G)** *DPAGT1* model flies crossed to a *Dop2R* overexpression line (UAS-Dop2R, BDSC 86134) had worse eye size. **(H)** A heterozygous null of *Dop1R1* (BDSC 92640) results in worse eyes in the *DPAGT1* model. **(I)** *Dop2R* RNAi (BDSC 36824) improved *DPAGT1* model [*Dop1R1*^*+/-*^] flies, but to a smaller maximal eye size than in *DPAGT1* model flies alone (indicated by dashed line, averaged from Fig 3D). All graphs are of female flies. See S2 and S3 Tables for more details. * p<0.05, *** p<0.001, **** p<0.0001 (Student’s t-test or One-way ANOVA with multiple comparison correction).

DA synthesis, transport, and downstream receptor signaling is well-conserved between *Drosophila* and humans [44,45] (Fig 3A). In this section, we primarily discuss female data, as females had an overall more robust response to manipulating DA signaling. All results, including male data, can be found in S2 and S3 Tables. Three DA receptor antagonists were suppressors with moderate Z-scores in our screen (Z-scores: 1.8–1.86, S1 Table). These included sulpiride, a D2/D3 receptor antagonist [46], paliperidone, an antipsychotic that antagonizes D2 and 5-HT2A receptors [47], and prochlorperazine, a first-generation antipsychotic that primarily antagonizes D2 receptors but also targets adrenergic, cholinergic, and histaminergic receptors [48–50]. Of these, validation testing of prochlorperazine improved the *DPAGT1* model at 1 μM in females (Fig 3B), and paliperidone also improved male flies (S2 Table). While evaluating our drug classes, we noticed that one D2 receptor antagonist—trifluoperazine [51]—had no observed progeny in the original screen (S1 Table). This is sometimes due to drug lethality like the insecticide fipronil (S1 Table), but it can also cause a false negative if parental flies did not mate well. Retesting trifluoperazine resulted in progeny and a robust increase in eye size in the *DPAGT1* model at the 5 μM dose in both sexes (Fig 3C and S2 Table). While prochlorperazine, paliperidone, and trifluoperazine overlap in function on D2 receptors, they can also target other receptors and pathways. Given this, we focused on DA signaling by genetically reducing the expression of their shared target *Dop2R* (human: *D2R*) in the *DPAGT1* model.

*Dop2R* is the only *Drosophila* ortholog of human genes *D2R*, *D3R*, and *D4R*. These encode for "D2-like" G-protein coupled receptors (GPCRs) that inhibit the adenylate cyclase/cAMP signaling pathway [44,45] (Fig 3A). Matching the pharmacological data from the primary screen, reduced expression of *Dop2R* using RNAi resulted in a strong improvement of eye size across all RNAi lines and replicates tested (Fig 3D and 3E and S3 Table), compared to other tested drug and RNAi treatments. In addition, their eyes showed strong qualitative improvement as they had fewer "glassy" eye sections (Fig 3E). We also crossed a heterozygous *Dop2R* null allele [52] into the *DPAGT1* model (note that *Dop2R* is X-linked and only females are analyzed here). Mimicking the RNAi, *DPAGT1* model flies carrying a heterozygous null *Dop2R* mutation also resulted in improved eye size (Fig 3F). This indicates that this improvement is not due to any indirect UAS-GAL4 [33] effects. Finally, overexpressing *Dop2R* [52] in the *DPAGT1* model decreased eye size (Fig 3G), confirming what is expected given the knockdown and null result. Genetically manipulating *Dop2R* had little effect on its own, with only two cases changing eye size: an increase in *Dop2R* OE females, and a minor decrease in *Dop2R* hemizygous null males (S3 Table). Overall, this indicates that reduction of D2 receptor signaling improves the *DPAGT1* model.

After release into the synaptic cleft, DA can bind to several receptors in the fly. This includes the Dop2R receptor as well as the Dop1R1, Dop1R2, and DopEcR receptors [44,45]. *Dop1R1* and *Dop1R2* are orthologs of the human *D1R* and *D5R* genes. These encode "D1-like" GPCRs that activate the adenylate cyclase/cAMP signaling pathway [44,45] (opposite of "D2-like" receptors). Given the opposite downstream effect of these receptors compared to *Dop2R*, we hypothesized that they would have an opposite effect on eye size. We crossed heterozygous null mutations of *Dop1R1* [53] and *Dop1R2* [52] into the *DPAGT1* model. Supporting our hypothesis, *DPAGT1* model flies carrying a heterozygous null *Dop1R1* mutation had worse eyes than the *DPAGT1* model alone (Fig 3H and S3 Table). *DPAGT1* model flies carrying a heterozygous null *Dop1R2* mutation had only a mild effect in one female replicate and no effect in males (S3 Table).

Given the strong partial rescue effect of *Dop2R* RNAi (Fig 3D–3E), we tested whether this loss of *Dop2R* could partially rescue the loss of *Dop1R1* in the *DPAGT1* model. We created a recombinant line of the *DPAGT1* model with the *Dop1R1* null mutation (*DPAGT1* model [*Dop1R1*^*+/-*^]). Loss of *Dop2R* does partially rescue the *DPAGT1* model [*Dop1R1*^*+/-*^] eye size, but to a lesser extent than loss of *Dop2R* on its own (Fig 3I, p<0.0001, Student’s t test, *Dop2R* RNAi compared across experiments). This result is in line with the opposing effects of *Dop1R1* and *Dop2R* on downstream cAMP signaling (Fig 3A).

The fourth DA receptor in *Drosophila*, DopEcR, has no clear human ortholog. Similar to Dop1R1, DopEcR activates the downstream adenylate cyclase/cAMP signaling pathway through DA binding. However, DopEcR can also activate the Mitogen-Activated Protein Kinase (MAPK) pathway through binding of the insect steroid ecdysone [44,45]. RNAi knockdown of *DopEcR* also improved eye size in the *DPAGT1* model (S3 Table). Taken together, these data suggest that loss of DA signaling through Dop2R and DopEcR is beneficial to the *DPAGT1* model, while loss of DA signaling through Dop1R1 is detrimental to the model.

### Impairing DA synthesis and recycling can improve the DPAGT1 model

**Fig 4:**
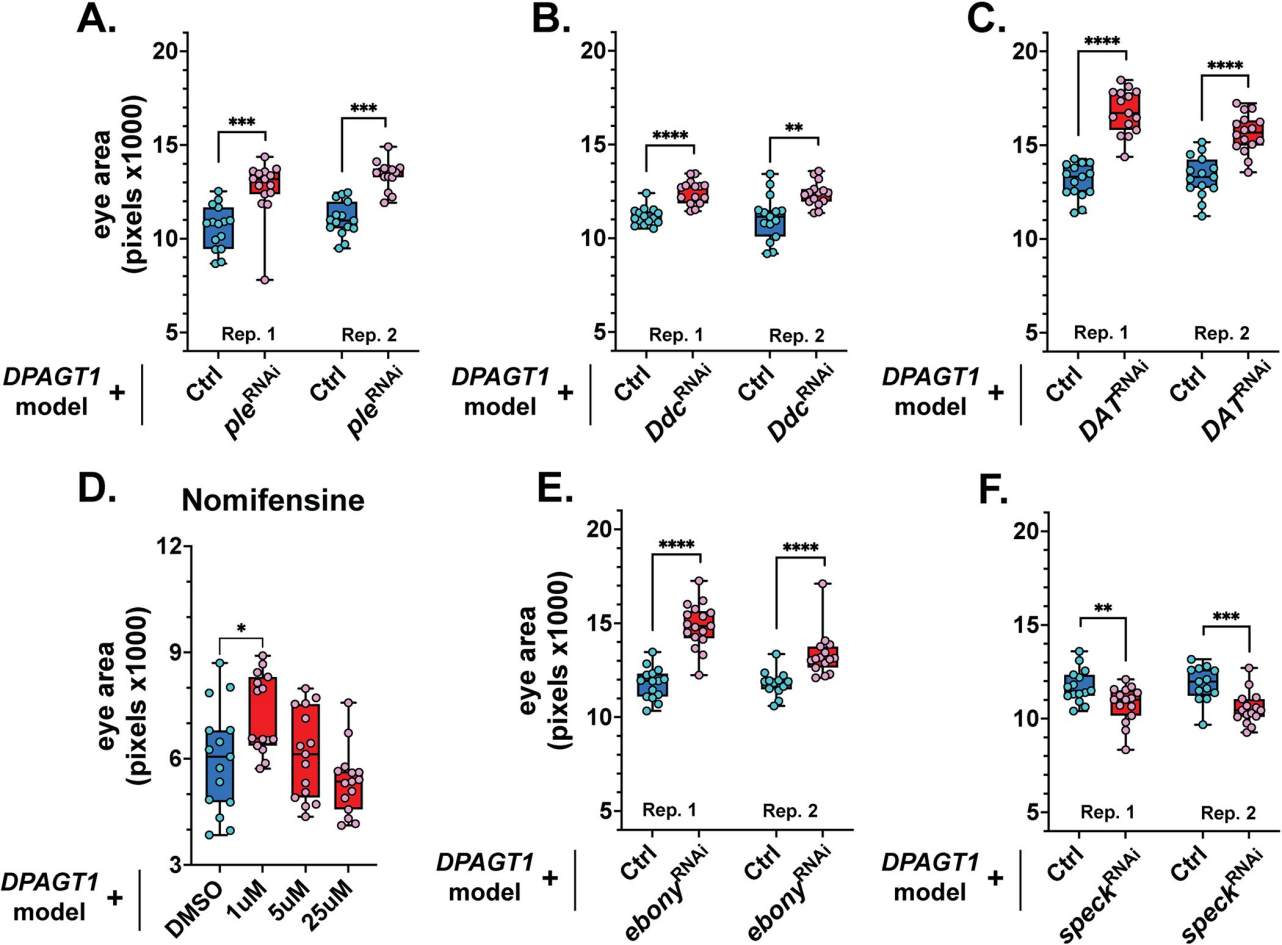
Inhibiting DA synthesis and recycling improves the *DPAGT1* model. **(A-B)** RNAi targeting the genes encoding the DA synthesizing enzymes ple and ddc (BDSC 25796 and 27030) improved the *DPAGT1* model. **(C-D)** RNAi against the gene encoding the DA recycling protein DAT (BDSC 50619), or pharmacologic inhibition of DAT via nomifensine at 1 μM, improved the *DPAGT1* model. **(E)** RNAi against the gene encoding the DA recycling protein ebony (BDSC 28612) improved the *DPAGT1* model. **(F)** RNAi against the gene encoding the N-acetyltransferase speck resulted in worse eyes in the *DPAGT1* model. All data are of female flies. See S2 and S3 Tables for male data and more details. * p<0.05, ** p<0.01, *** p<0.001, **** p<0.0001 (Student’s t-test or One-way ANOVA with multiple comparison correction).

DA is synthesized by the enzymes tyrosine hydroxylase and DOPA decarboxylase, which are encoded by the *ple* and *Ddc* genes, respectively (humans: *TH* and *DDC*). DA is then transported into vesicles by the vesicular monoamine transporter VMAT, encoded by *Vmat* (humans: *VMAT2*), before being shuttled into the synaptic cleft [44,45] (Fig 3A). Similar to knockdown of *Dop2R*, knockdown of the first DA synthesis gene, *ple*, and the second DA synthesis gene, *Ddc*, strongly improved the *DPAGT1* model (Fig 4A and 4B and S3 Table). Knockdown of *Vmat* also improved the model, but it had a similar increase on its own (S3 Table). Overall, this suggests that inhibiting DA synthesis is beneficial to the *DPAGT1* model.

There are several ways that DA can exit the synaptic cleft. DA can be recycled back from the synaptic cleft to the presynaptic neuron by the DA transporter DAT [44,45] (encoded by *DAT*) (Fig 3A). Knockdown of *DAT* strongly improved *DPAGT1* model eye size, matching the same increase as loss of *Dop2R* (Fig 4C, S3 Table). The DAT inhibitor [54,55] nomifensine maleate had a positive Z-score in the primary screen (0.84) but did not reach our hit threshold. Because we saw strong improvements in eye size when *DAT* was knocked down compared to other treatments, we tested a version of this drug lacking the maleate salt (S2 Table). In line with the genetic data, treatment with nomifensine improved the *DPAGT1* model at 1 μM (Fig 4D). In *Drosophila*, but not in humans, DA can also be converted to the metabolic product NBAD for translocation and conversion back into DA. These steps are performed by the enzymes black, ebony, and tan through the β-alanylation pathway [44,45] (encoded by *black*, *ebony*, and *tan*) (Fig 3A). ebony and tan convert and shuttle DA, while black creates the β-alanine necessary for ebony to function [44]. RNAi knockdown of the first β-alanylation gene, *ebony*, strongly improved the *DPAGT1* model (Fig 4E and S3 Table). However, while knockdown of *black* improved the *DPAGT1* model, it had a similar increase on its own (S3 Table). There are no available RNAi lines for *tan*. Overall, inhibiting the recycling of DA back into the synaptic cleft through DAT or ebony improves the *DPAGT1* model.

In contrast to these recycling pathways, DA can exit the synaptic cleft through metabolism into the inactive compound NADA in *Drosophila*. This occurs through the N-acetyltransferase enzyme, speck, and prevents further signaling by that DA molecule. While speck has no clear human ortholog, it serves a similar role to monoamine oxidases in humans [44]. Because speck removes DA, we hypothesized that speck would have the opposite effect of the DA synthesis genes. Supporting this hypothesis, knockdown of *speck* resulted in worse eye sizes in the *DPAGT1* model (Fig 4F and S3 Table). Thus, inhibiting the removal of DA from the synaptic cleft is detrimental to the *DPAGT1* model.

Generally, these results fit the hypothesis that most *DPAGT1* model outcomes can be predicted by how a treatment affects DA binding to Dop2R (Fig 3A). For example, inhibiting DA synthesis or recycling may improve the *DPAGT1* model by reducing the flux of DA to Dop2R. In contrast, because speck normally removes DA and reduces its binding to Dop2R, this could explain why inhibiting speck worsens the *DPAGT1* model. Overall, these data strongly link the inhibition of DA synthesis, recycling, and signaling with improvement of *DPAGT1* deficiency. Drugs that impair D2 signaling or synthesis, or are agonists of D1 receptors, may be good therapeutics for DPAGT1-CDG.

### Histaminergic signaling is beneficial to the DPAGT1 model

**Fig 5:**
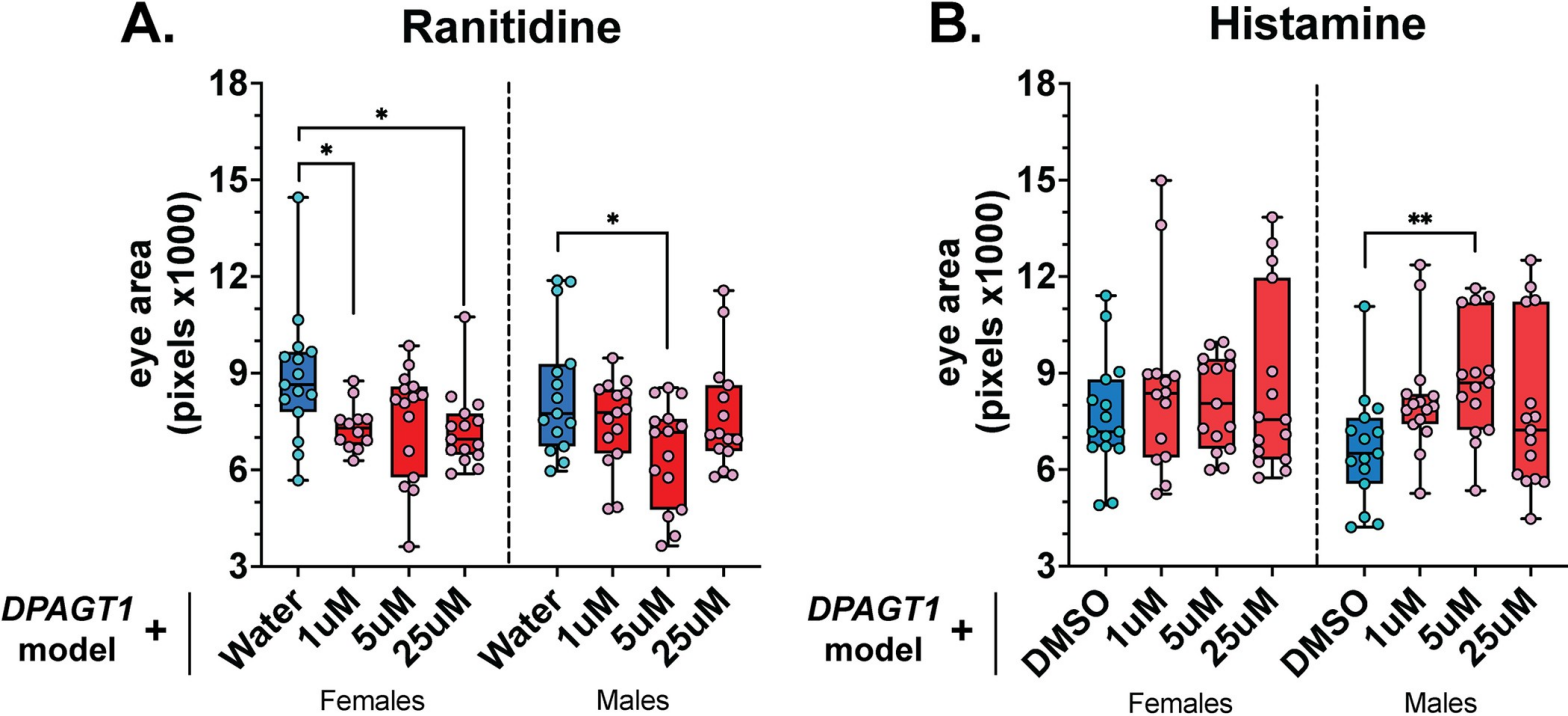
Antagonizing histamine signaling worsens the *DPAGT1* model. **(A)** The H2 antagonist Ranitidine worsens the *DPAGT1* model at multiple concentrations. **(B)** The H1/H2 agonist histamine can partially rescue the *DPAGT1* model at 5 μM in males. See S2 Table for more details. * p<0.05, ** p<0.01 (One-way ANOVA with multiple comparison correction).

The Histamine 2 (H2) receptor antagonist ranitidine, used to decrease stomach acid [56], was a strong enhancer in our screen (Z-score = -2.33, S1 Table). Histamine and DA have some overlapping biology. For example, *Drosophila* can process DA and histamine using the same β-alanylation enzymes—ebony, black, and tan [44,57,58]. In addition, loss of histamine can upregulate depolarization-stimulated DA release and receptor expression in mice [59]. Given the impact of DA signaling on the *DPAGT1* model (Figs 3 and 4), our results from *ebony* knockdown (Fig 4E and S3 Table), and the finding of ranitidine, we tested histaminergic signaling further. In line with the primary screen, ranitidine significantly worsened eye size in the *DPAGT1* model at multiple doses (Fig 5A). As ranitidine is an H2 receptor antagonist, we tested if exogenous histamine (an H1/H2 agonist) would have the opposite effect. Treatment with histamine showed statistically significant increase at 5 μM in males and increased the upper distribution of both sexes at 25 μM (Fig 5B). Histamine is an atypical medication which is used in select cancer therapies [60]. Histamine could represent a new therapeutic for patients, and these data also suggest that some antihistamines may be detrimental under *DPAGT1* impairment.

### Inhibiting the ion transporter NKCC1 improves the DPAGT1 model

The loop diuretic bumetanide was a strong suppressor hit in our screen (Z = 2.04). Bumetanide inhibits the ion cotransporters NKCC1 and NKCC2 (encoded by *NKCC1* and *NKCC2* in humans). Both proteins transport Na+, K+, and Cl- ions into cells primarily in the secretory epithelia or renal tissues, respectively [61]. In addition to its diuretic property, bumetanide has recently been tested as an off-label anti-seizure medication to some success [62–65]. Finally, we previously found that NKCC1 is a genetic modifier of NGLY1 deficiency, another CDG [66].

**Fig 6:**
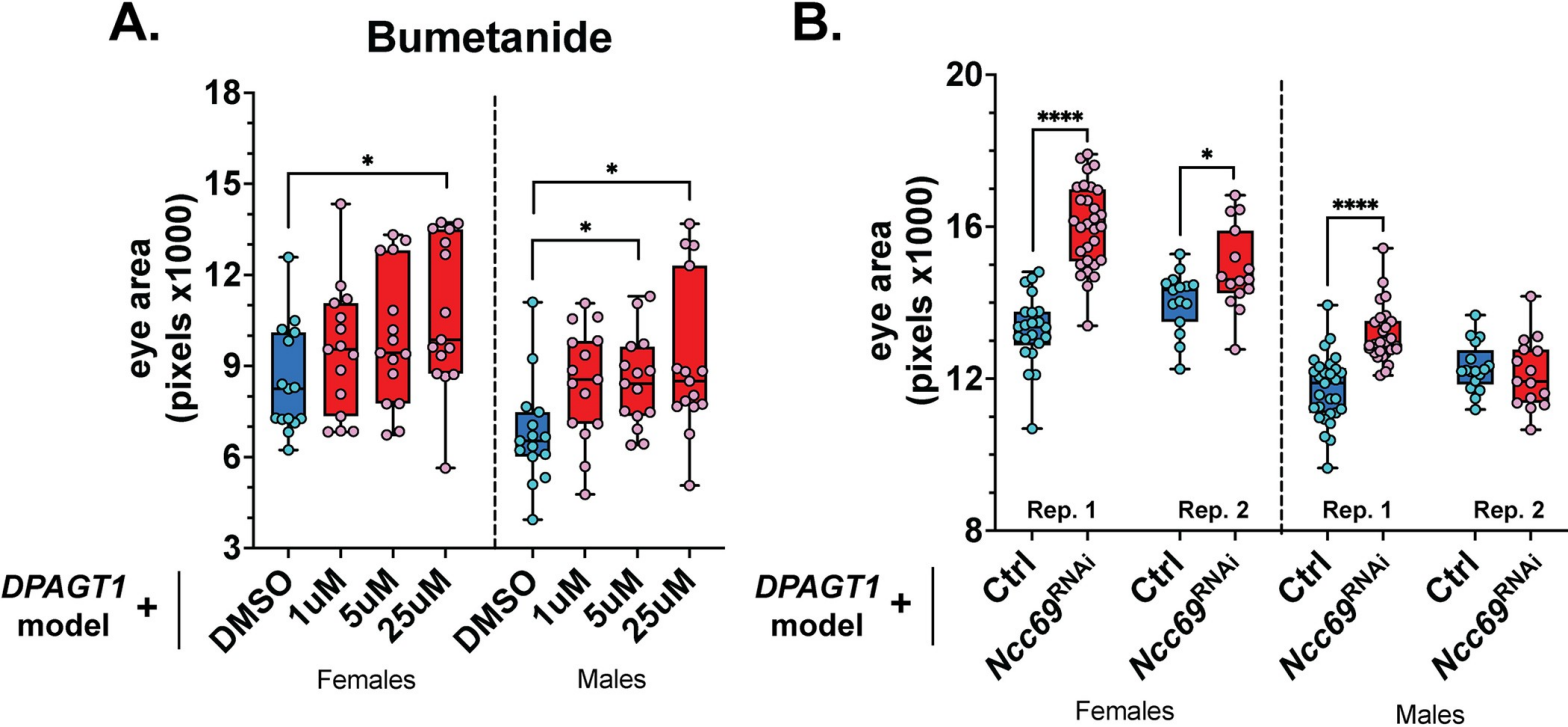
Inhibiting the ion transporter NKCC1 improves the *DPAGT1* model. **(A)** Bumetanide increases eye size in the *DPAGT1* model. **(B)** Genetic knockdown of *Ncc69*, which encodes the target of bumetanide inhibition, also increases eye size in the *DPAGT1* model. See S2 and S3 Tables for more details. * p<0.05, **** p<0.0001 (Student’s t-test or One-way ANOVA with multiple comparison correction).

Matching the primary screen, bumetanide caused a strong increase in eye size in the *DPAGT1* model in a dose-dependent manner (Fig 6A). *Ncc69* is the *Drosophila* ortholog of *NKCC1* and *NKCC2* [66,67]. Genetic knockdown of *Ncc69* also resulted in an increase in eye size in the *DPAGT1* model (Fig 6B and S3 Table). This corroborates with the bumetanide data that inhibiting NKCC1/NKCC2 activity is beneficial under *DPAGT1* deficiency. Thus, drugs that inhibit NKCC1 activity may be beneficial when *DPAGT1* activity is reduced.

### COX inhibitors improve the DPAGT1 model

**Fig 7:**
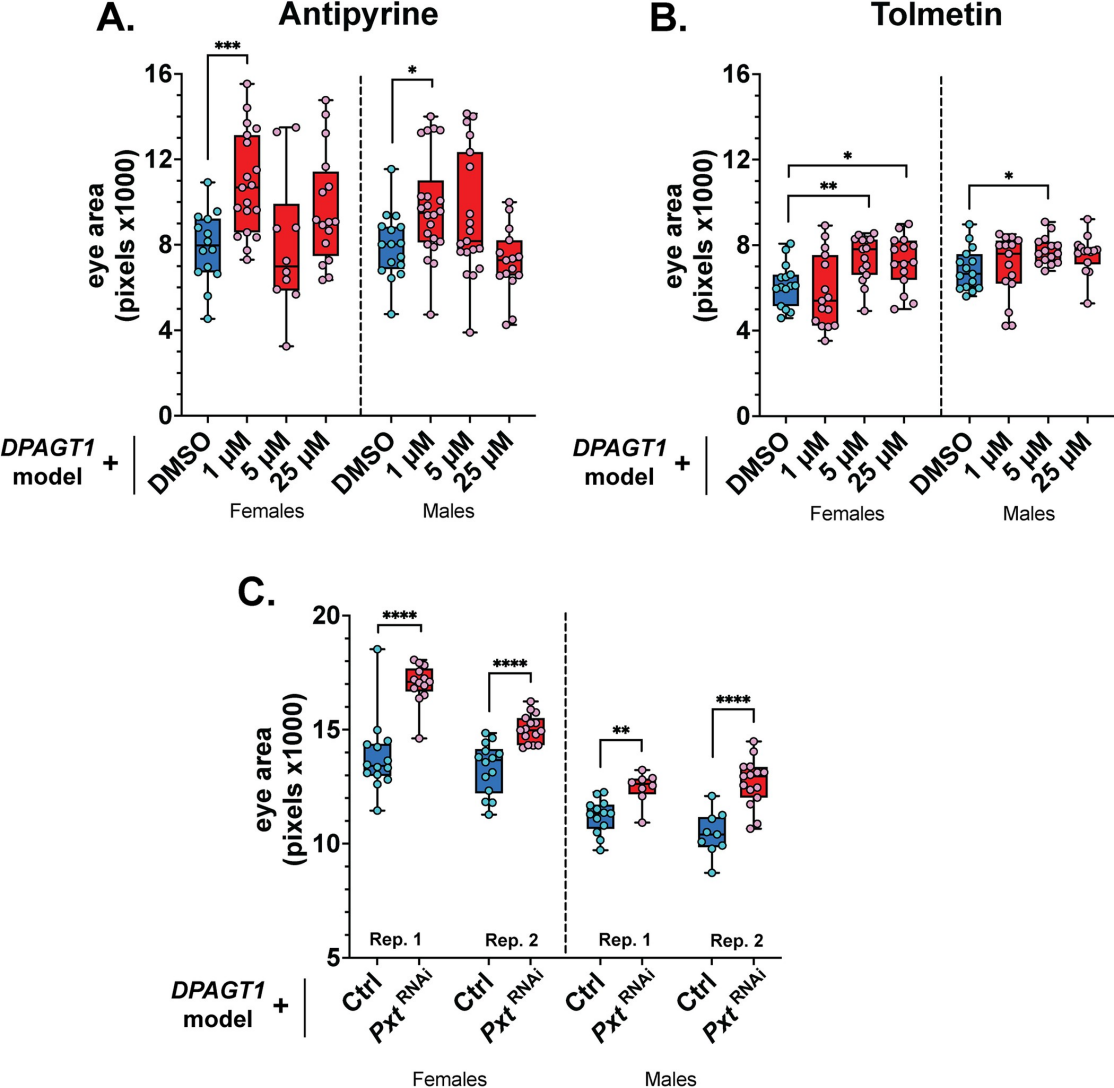
Inhibiting COX enzymes improves the *DPAGT1* model. **(A-B)** Both antipyrine and tolmetin improved the eye size of the *DPAGT1* model. **(C)** RNAi against the gene encoding the COX-like enzyme Pxt (BDSC 32382) partially rescued the *DPAGT1* model. See S2 and S3 Tables for more details. * p<0.05, ** p<0.01, ** p<0.001, **** p<0.0001 (Student’s t-test or One-way ANOVA with multiple comparison correction).

The cyclooxygenase signaling pathway involves the synthesis of prostaglandins that are important for processes such as inflammation and growth [68–70]. Many non-steroidal, anti-inflammatory drugs (NSAIDs), such as ibuprofen, inhibit the first enzymes in this process—cyclooxygenases COX-1 and COX-2 (encoded by *COX1* and *COX2* in humans). There were three COX-1/COX-2 inhibitor hits in our screen: triflusal [71], antipyrine, and the antipyrine metabolic product, 4-hydroxyantipyrine [72]. Of these, antipyrine significantly increased average eye size at the 1 μM dose, and multiple antipyrine doses resulted in a positive shift in *DPAGT1* model eye size distributions (Fig 7A).

NSAIDs are commonly used and would be easy to access for patients [73]. However, COX inhibitors can vary in their specificity and degree of COX inhibition, and this can affect their efficacy and tolerance [74–77]. As such, we tested five other COX inhibitors using the *DPAGT1* model. Of these, the COX-1/COX-2 inhibitor tolmetin [78] significantly improved the *DPAGT1* model at 5 μM in both sexes, and at 25 μM in females (Fig 7B). The *Drosophila* gene *Pxt* is the ortholog of human *COX1* and *COX2* [79,80]. Similar to antipyrine and tolmetin, knockdown of *Pxt* also resulted in improved eye size in the *DPAGT1* model (Fig 7C and S3 Table). Given that inhibiting *Pxt* is beneficial, we hypothesize that antipyrine and tolmetin specifically improve the *DPAGT1* model because they may be more efficacious or well-tolerated under *DPAGT1* deficiency. Overall, inhibiting prostaglandin signaling through COX inhibition is beneficial to the *DPAGT1* model, and certain COX inhibitors may be useful in alleviating symptoms of *DPAGT1* impairment in patients.

## Discussion

**Fig 8:**
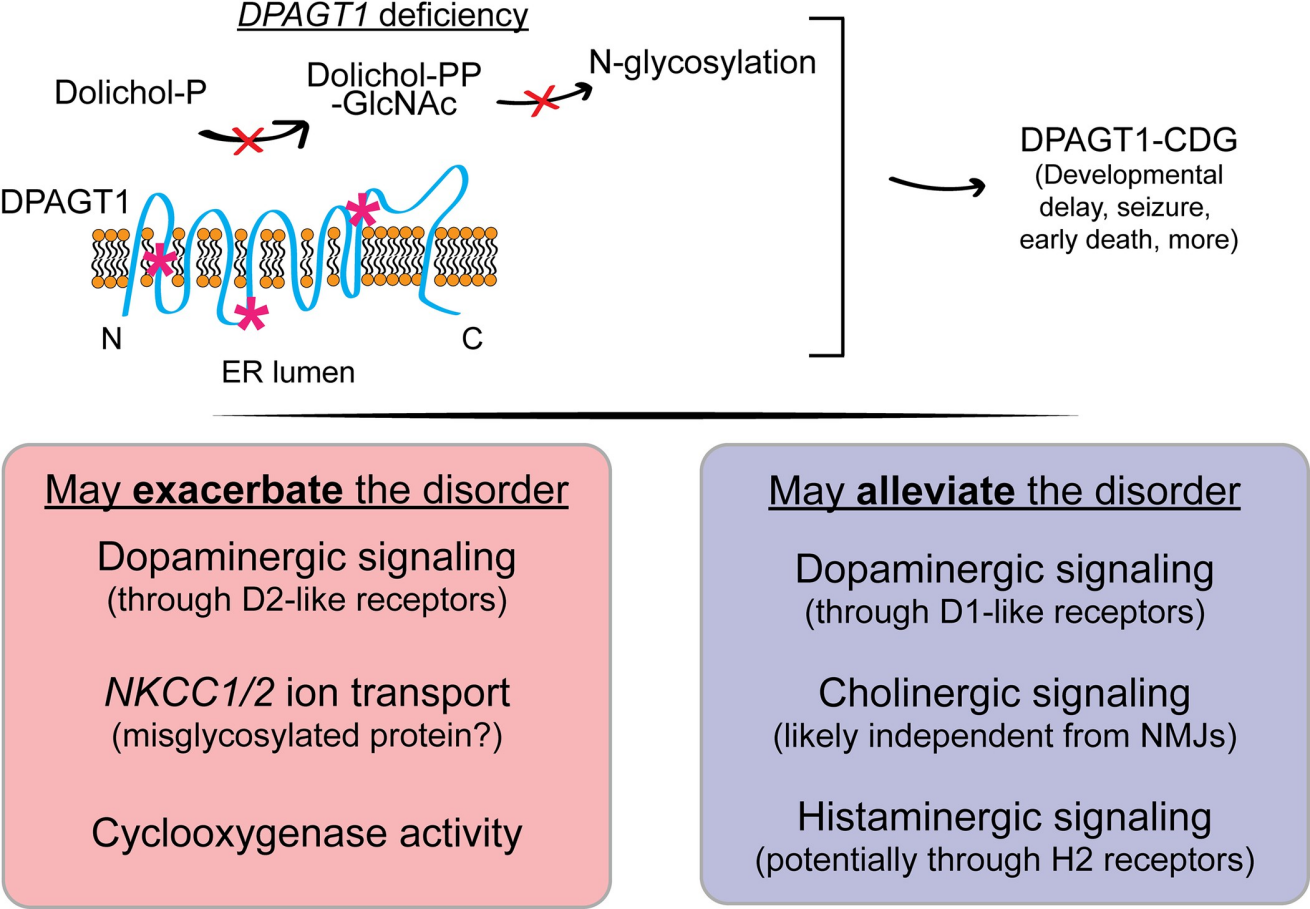
New connections to DPAGT1 biology. DPAGT1-CDG is caused by mutations in the enzyme DPAGT1. Here we summarize several new biological connections to DPAGT1 discussed throughout, along with potential specifics to each individual pathway in parentheticals.

Here, we identify multiple drugs that improve a *Drosophila* model of *DPAGT1* deficiency. Overall, 8/21 (38%) drugs tested from the primary screen validated in further dose-response analysis. In addition, 6/16 (38%) drugs derived from the primary screen improved the *DPAGT1* model. Almost all tested drugs were genetically validated by manipulating their target genes in *Drosophila*. We found that drug classes involving acetylcholine signaling, dopamine (DA) synthesis, histamine receptors, NKCC1/2, and cyclooxygenase inhibition might be good candidates for treating DPAGT1-CDG (Fig 8).

One of the enriched classes was acetylcholine (ACh)-related drugs. Identifying this drug class provides strong validation of our fly model and drug screen, as this class is already used by both DPAGT1-CDG and -CMS patients [17,18,42]. In addition, multiple AChE inhibitors currently taken by patients improved the *DPAGT1* model. This suggests that our CDG fly model is a reasonable representation of the disorder, and that these models are valuable tools for identifying potential therapeutics. We found that genetically or pharmacologically inhibiting acetylcholine breakdown is beneficial under *DPAGT1* impairment. Acetylcholinesterase inhibitors are taken to improve muscle function in *DPAGT1* patients by increasing acetylcholine signaling at neuromuscular junctions (NMJs) [81]. However, in *Drosophila*, glutamate is the excitatory neurotransmitter in NMJs [82], and the fly eye is likely not impacted by NMJ signaling regardless. Outside of NMJs, acetylcholine can also bind to nicotinic acetylcholine receptors to mediate DA release in flies [83] and mammals [84,85]. Given that increased DA appears negative for the model, it is possible that the benefit of increased acetylcholine signaling counteracts any negative consequences of increased DA release. Alternatively, acetylcholine-mediated DA release may be more prevalent in cells expressing higher levels of D1-like DA receptors, which would prove beneficial.

Acetylcholine is important for many biological processes, including neurodevelopment [86–88] and growth [89–91]. Given the impaired eye development in the *DPAGT1* model, these processes may underlie the improvement from *ACE* inhibition (and the ostensible increase in acetylcholine). While muscle weakness in patients and the eye defect in the *DPAGT1* model are distinct phenotypes, they are connected because both are improved by decreasing breakdown of acetylcholine. It is possible that impaired acetylcholine signaling may occur outside of muscles in DPAGT1-CDG patients. For example, patients also have impaired neurodevelopment as well as eye disorders such as cataracts [20,92]. Further use of AChE inhibitors may be worth exploring further in patients given the improvement in eye development in our model. Overall, acetylcholinesterase inhibitors have beneficial effects under *DPAGT1* impairment in flies and humans.

We found that inhibiting Dop2R signaling improves the *DPAGT1* model. In addition, knockdown of DA synthesis or recycling genes improves the model. Thus, the synthesis of DA, and its binding to the Dop2R, are detrimental under *DPAGT1* inhibition. Dop2R signaling inhibits the downstream adenylate cyclase/cAMP signaling pathway. While cAMP signaling has many diverse functions, it is important for proper neuronal connectivity and survival [93,94]. Therefore, inhibiting Dop2R signaling may increase cAMP signaling and allow for improved neuronal outcomes. This could underlie the benefits in the *DPAGT1* model from Dop2R genetic and pharmacological inhibition. Supporting this hypothesis, heterozygous knockout of the Dop1R1 receptor worsened the *DPAGT1* model. Binding to Dop1R1 may counteract signaling to Dop2R through increased cAMP signaling. Given this, drug classes such as D2R antagonists or D1R agonists might be good therapeutics for DPAGT1-CDG. Of note, we identified two monoamine oxidase inhibitors (MAOIs) as enhancers in the screen (moclobemide and selegiline, S2 Table). MAOIs inhibit MAOs to prevent the breakdown of neurotransmitters such as DA [95]. It is possible that these MAOIs were enhancers because they increased the amount of DA in the presynaptic neuron, in line with our data on DA synthesis and recycling.

Misregulated DA signaling is associated with neurological disorders such as Parkinson’s disease, schizophrenia, and bipolar disorder [96]. DPAGT1-CDG has no previous clear connection to DA. However, both DPAGT1-CDG and -CMS patients have abnormal gait [42,97]. DPAGT1-CDG patients also have neurological symptoms including seizures and intellectual disability [6,14]. Misregulated DA could underlie some symptoms of DPAGT1-CDG. Interestingly, the DA pathway is misregulated in a fly model of another CDG, NGLY1 deficiency [25]. Further, aripiprazole, a complex agonist of D2 receptors [98], could rescue worm and fly disease models of NGLY1 deficiency [99]. Thus, DA signaling may be important in glycosylation disorders more generally.

Inhibiting the ion transporter Ncc69 via bumetanide or RNAi resulted in a strong improvement of the *DPAGT1* model compared to other treatments. In humans, NKCC1 has three sites of N-glycosylation while NKCC2 has two (via Uniprot [100]). We previously found that loss of the deglycosylating enzyme NGLY1 results in reduced NKCC1 activity [66], and other studies find that inhibiting N-glycosylation reduces the function of NKCC1 and NKCC2 [101,102]. Therefore, proper glycosylation or deglycosylation of NKCC1 and NKCC2 is critical to their function. Given this, impaired N-glycosylation in the *DPAGT1* model likely impairs the function of Ncc69. Thus, it was surprising that further reducing *Ncc69* expression by RNAi actually improved the *DPAGT1* model. We hypothesize that under *DPAGT1* impairment, any Ncc69 that is expressed may be misglycosylated and subsequently may be misfolded, or have aberrant function, and contribute to disease pathogenesis.

Bumetanide is typically used as a loop diuretic in patients with edema and hypertension [65] due to its inhibition of NKCC2 in renal tissue. However, bumetanide also inhibits NKCC1 which is expressed more systemically. This includes neurons where NKCC1 affects cell polarization and levels of GABA that are important for proper CNS function [103–105]. To that end, bumetanide has been used more recently as an anti-seizure medication in mice [104] and off-label in humans [105]. In addition, oral solutions of bumetanide were recently used in clinical trials for autism in children [106]. However, bumetanide still impacts renal function when used for neurological symptoms, causing diuretic side effects [107]. These side effects are likely more challenging for those who are already medically fragile such as DPAGT1-CDG patients. However, the structural basis for bumetanide inhibition was recently described [65], and this could pave the way for future bumetanide-like drugs. It is possible that such drugs could be used as anti-seizure medications without the diuretic side effects. Given that bumetanide improved the eye development of the *DPAGT1* model, it also suggests a role for bumetanide-like drugs beyond their use as anti-seizure medications.

The COX inhibitors tolmetin and antipyrine improved the *DPAGT1* model. While now discontinued in the US (via PubChem [108]), tolmetin was used for decades as an alternative to aspirin and has similarly few side effects [109]. Antipyrine, also known as phenazone, is an antipyretic currently used to treat ear infections [110]. In the past, antipyrine was used orally for decades and was one of the earliest prescribed analgesics [111]. Interestingly, while the antipyrine metabolite, 4-hydroxyantipyrine, was a hit in the screen, it did not validate in our hands. This could indicate some volatility of this metabolite or that a different dose is required. Inhibiting the cyclooxygenases COX-1 and COX-2 is a common drug mechanism for alleviating inflammation and pain [68,69]. However, there is also evidence for the use of COX-1 and COX-2 inhibitors in treating cognitive deficits and convulsions [70,112,113], both of which are relevant to DPAGT1-CDG. While only two NSAIDs improved our model, this category may still be promising for DPAGT1-CDG given their high usage and ease of patient access [73].

We tested multiple drug doses to determine if some doses could provide better partial rescue of our model compared to our initial screen of 5 μM. In our model, some drugs may need high concentrations to elicit a phenotype (e.g. bumetanide), while others may have decreased efficacy at too high of a dose (e.g. nomifensine). This is a common finding in dose-response analyses [114]. Ultimately, as long as one dose worked, we considered it as a hit because many drugs require specific dose ranges to be effective [114]. We also found several drugs that partially rescue one sex stronger than the other (e.g. acetylcholinesterase inhibitors). *Drosophila* are sexually dimorphic and have differences in behavior, development, and immunity, among other pathways [115–117]. In addition, previously studied drugs such as rapamycin are only effective in one sex [118]. We chose to perform validation in both sexes to reduce potential sex-specific effects. In each drug category, at least one pharmacological or genetic treatment improved each sex. Thus, while there may be some sex differences in efficacy, we did not find any completely sex-dependent biological pathways affecting the *DPAGT1* model.

Around 20% of all proteins are glycosylated [119], and membrane proteins (the targets of most drugs) are almost all glycosylated [120]. Given the role of DPAGT1 in N-glycosylation [6], it is possible that the glycosylation status of drug targets could bias our hits. Many of our suppressor drugs target proteins that are N-glycosylated. This includes acetylcholinesterase [121], DA receptors [122,123], DAT [124], NKCC1 [101], and COX-1/COX-2 [125]. However, it also includes acetylcholine receptors [38], which would be activated by acetylcholinesterase inhibitors and ostensibly be beneficial for the *DPAGT1* model. In addition, the validated enhancer, ranitidine, targets the H2 receptor which has a putative N-glycosylation site (via UniProt [100]). Finally, three suppressors did not validate, but also target N-glycosylated receptors: this includes melatonin receptors [126], NMDA receptors [127], and adrenergic receptors [128,129] (S2 Table). While it is possible that the N-glycosylation status of a particular targeted protein is important for a specific drug hit, we found no general correlation between N-glycosylation status and suppressor or enhancer hits.

In this study, we screened 1,520 small molecules and identified multiple drugs that improved a *Drosophila* model of DPAGT1-CDG. We verify these findings using pharmacologic and genetic manipulation which strongly align with the known mechanisms of these therapeutic hits. We establish new biological connections between DPAGT1 and DA signaling, NKCC1, and prostaglandin synthesis. These findings may help create new treatment options for DPAGT1-CDG, and our validated drug hits represent potential therapeutics for patients.

## Materials and methods

### Fly stocks and maintenance

All flies were maintained at room temperature. All experiments were performed in a 20°C incubator unless otherwise noted. Flies were fed standard Glucose medium (D2) from Archon Scientific (Durham, North Carolina). We used fly stocks from the Bloomington Drosophila Stock Center and the Vienna Drosophila Resource Center [130] (listed in S3 Table). The *DPAGT1* model (*eya* composite-GAL4, UAS-*Alg7* RNAi [III]) was described previously and used the BDSC *DPAGT1* RNAi stock 53264 (TRiP.GLC01825) [28]. Its genetic background control, *eya* composite-GAL4 (III), was a gift from Justin Kumar (Indiana University Bloomington). As described previously, we do not use the common GMR-GAL4 eye driver, as it is expressed later in development and did not result in a rough eye phenotype [28].

The second model of *DPAGT1* deficiency (S1 Fig) was generated using BDSC *DPAGT1* RNAi stock 51869. This stock was crossed to the *eya* composite-GAL4 (III) driver and balanced using the same Chr. III balancer, [TM3, Dfd-YFP, *Sb*], as the original *DPAGT1* model. This resulted in *w-*, *y-*, *v-*, *sev-;* P[y+, v+, *Alg7* RNAi]); (*eya composite*-GAL4, w+/TM3, Dfd-YFP, *Sb*) flies.

The *DPAGT1* model containing the *Dop1R1* null mutant (BDSC 92640) recombination (*DPAGT1* model [*Dop1R1*^*+/-*^*]*) was generated as follows. We crossed the *DPAGT1* model [28] to the *Dop1R1* null mutant (BDSC 92640) to create (*eya composite*-GAL4, w+, P[sc+, y+, v+, *Alg7* RNAi])/(*Dop1R1* null) (III) animals. We then crossed this line to a balancer line containing *D*/(TM3, *ser*) (III) and examined progeny for crossover events. We collected progeny with smaller eye size than the *DPAGT1* model and *ser*. We then replaced the (TM3, *ser*) balancer with (TM3, *Sb*), self-crossed the line to ensure stability of the phenotype, and refer to this line as *DPAGT1* model [*Dop1R1*^*+/-*^] flies (*eya composite*-GAL4, w+, P[sc+, y+, v+, *Alg7* RNAi], *Dop1R1* null)/(TM3, *Sb*) (III).

### RNA processing and qPCR sequencing

We collected 8–10 heads of 2–7 day-old female *Drosophila* of the *eya* composite-GAL4 background strain and the *DPAGT1* model. We placed heads directly into 100 μL TRIzol reagent in eppendorf tubes, manually dounced the heads for 20 strokes using a plastic douncer, then placed these tubes onto ice. We then stored these samples for at least 24hrs at -80oC. We processed the TRIzol samples into RNA using the Zymo Direct-zol RNA Miniprep kit (Zymo Research cat. R2061), and we included the DNAseI treatment step. We converted RNA into cDNA using the ProtoScript II First Strand cDNA Synthesis Kit (NEB cat. E6560L). We then used this cDNA, PowerUp SYBR Green Master Mix (ThermoFisher cat. A25741), and forward/reverse primers to perform qPCR analysis on the QuantStudio 3 (ThermoFisher cat. A28567). Primers used: DPAGT1 F: ACTTCATGCTGCCTTTCCTG, DPAGT1 R: AAGTCATGCCGGCAAAGTAG; RpL19 F: AGGTCGGACTGCTTAGTGACC, RpL19 R: CGCAAGCTTATCAAGGATGG.

### *in vivo* small molecule screen

For the primary screen, we used the Prestwick Chemical Library (PCL, Illkirch, France). The PCL contains 19 plates of 80 compounds each (1,520 total). All compounds came dissolved in DMSO at a concentration of 10 mM. Compounds were diluted further to 1 mM in phosphate-buffered saline. To make food containing these compounds, we used 500cc bags of standard Glucose medium (D2) from Archon Scientific (Durham, North Carolina). We dispensed this media into a beaker, microwaved until it liquified, then allowed it to cool on a heated stir plate under agitation. Once reaching 60°C, 1 mL of this media was dispensed into vials containing aliquots of the dissolved drugs or DMSO to reach a final concentration of 5 μM and 0.05% DMSO. We used up to eight DMSO control vials for each plate of 80 unique drugs.

Once the food was cooled, we placed 3–5 premated *DPAGT1* model intercrossed females and 2–3 males into each vial. Flies were allowed to lay eggs for 1–4 days until visual inspection indicated that approximately 30 eggs were laid. Flies were then removed. The *DPAGT1* model is balanced by TM3, *Sb* [28], and homozygotes are infrequent and semi-lethal. As such, we only collected heterozygote flies by selecting for the *Sb* phenotype. We collected up to five (average = 4.4, males and females), 2–7 day old progeny flies from each vial and froze them down at -80°C for later imaging. Drug names were masked, so both preparation of vials and collection of flies was done blinded to each drug.

Progeny eyes were imaged at 3x magnification (Leica EC3 camera). We determined eye area as previously described [131]. We masked image file names to blind observers to each treatment used. Within each set of 80 vials, we compared drug-treated animal eye sizes to the average of up to 8 DMSO controls and determined Z-scores (S1 Table). In one plate, drug-treated animals were instead compared to the plate average due to a technical error in the control (plate 03, S1 Table), but these values were in line with the rest of the plates. To determine if a drug was a hit, we used a Z-score threshold of 1.5 (derived from other *Drosophila* screens [132–134]).

We excluded all vials with a positive Z-score that contained only one fly (13 total vials, 0.9% of all 1,520 vials). 15 compounds were excluded for technical reasons (e.g. incorrect food pours, 1%). An additional 16 vials had eggs laid without any eclosed flies (1.1%). If ≤1 males were observed, but females were present, females were measured and compared to DMSO-treated females. This occurred in 47 vials (3.1%), and none of these reached our Z-score threshold. All reported Z-scores were derived from a single sex, and we indicate when females were scored in S1 Table.

### Drug and RNAi validation

For making drug validation food, we used the same method as the primary screen, except that we used 10 mL of media, and we used drug concentrations of 1 μM, 5 μM, and 25 μM (as done previously [25]). We chose this more "conservative" dose curve as these drugs were already hits at 5 μM. Because there may be slight differences in drug concentrations from vendors or during food preparation, we considered drugs to validate if any of the three concentrations improved eye size (after multiple comparison correction). Most compounds were dissolved in DMSO. However, we employed other solvents if needed to reach the maximum concentration of 25 μM (S2 Table). We validated using at least two compounds from each drug category (S2 Table) from the Prestwick Chemical Library by using secondary vendors to limit quality control errors. These vendors were either Cayman Chemical (Ann Arbor, MI), MedChemExpress (Monmouth Junction, NJ), or Sigma-Aldrich (St. Louis, MO). Specific compounds and their vehicles are listed in S2 Table.

We crossed each fly knockout, overexpression, or RNAi line to either the *DPAGT1* model or its control, w-;;*eya* composite-GAL4 (S3 Table). All RNAi in this study is driven by the *eya* composite-GAL4 line. RNAi or overexpression crosses used either attP40 (BDSC 36304), attP2 (BDSC 36303), or attP (VDRC 60100) as control comparisons, and null crosses used *w*^1118^ (VDRC 60000). Because the *DPAGT1* model uses RNAi endogenously, RNAi crosses resulted in double knockdown flies. When fly stocks were available, we used two different RNAi lines to reduce potential reagent-specific effects. Complete information on lines used can be found in S3 Table. The average N across every drug validation experiment was 14.5 flies (based on 3,186 fly measurements).

Both drug and RNAi validation used the same methodology as the primary screen for collecting animals and measuring eye sizes. Percentages listed in the text and S2 and S3 Tables represent the percent change of the treated mean compared to the control mean. See S4 Table for these raw data.

### Statistics

For group comparisons, data were analyzed by One-way ANOVA with Welch’s correction and the post hoc Dunnett’s test to account for multiple comparisons. For individual comparisons, data were analyzed by Student’s t test with Welch’s correction. We used GraphPad Prism v10 or Microsoft Excel for these analyses.

## Supporting information

S1 Table List of all drugs tested in drug repurposing screen. Each plate (first ##, 01–19) was tested independently of each other plate. See Methods for the Z-score calculation. "Females measured" means there were not enough males to measure, so females were measured instead. Excluded note meanings: "N = 1" means there was only one male (and one or no female) to measure; "No flies observed" means we did not observe any eclosed flies; "Technical reasons" means something unrelated to the drug negatively impacted the vial, such as an incorrect food pour.
(XLSX)

S2 Table Information on drug validation experiments. Tab information: "Primary hits" lists all drugs that reached at least 1.5 or -1.5 from the drug repurposing screen. "Rescuing drugs" lists all drugs that improved the *DPAGT1* model (whether directly or derived from the screen). This includes the quantitative results of what % they improved eye size over vehicle-treated *DPAGT1* model flies and their p-value (see Methods for statistics). The low/medium/high doses can be found in the tab "Compound sources, vehicles". "Non-rescuing drugs" lists drugs that worsened the model or had no effect. "Categories of tested drugs" lists the general drug class that each tested drug falls into. " Compound sources, vehicles" lists each drug tested during validation, where they were sourced from (including product numbers), their vehicle used, and doses used for each drug. Cells are labeled blue to indicate statistically significant positive changes, red represents negative changes, and gray represents non-significant changes.
(XLSX)

S3 Table Information on gene validation experiments. Quantitative results on the effect of genetic manipulations used on the *DPAGT1* model, *eya* composite-GAL4 control, or *DPAGT1* model [Dop1R1+/-]. This includes the specific stock number, the *Drosophila* gene affected, the nature of that stock (RNAi = RNA interference, OE = overexpression), the % change in eye size in each replicate, and the associated p-value (see Methods for statistics). Cells are labeled blue to indicate statistically significant positive changes, red represents negative changes, and gray represents non-significant changes. Each tab separates female and male experiments.
(XLSX)

S4 Table Drug and RNAi validation raw data. This file contains the raw pixel values of eye sizes of experimental and control conditions/genotypes for the validation experiments.
(XLSX)

S1 Fig Knockdown in the *DPAGT1* model and images of a second knockdown model. (A) Graph of *DPAGT1* knockdown in the *DPAGT1* model, * p<0.05, (Student’s t-test). (B) Representative images of a second *DPAGT1* knockdown model using the BDSC 51869 stock.
(PDF)

S2 Fig Representative images of female fly stocks. This is a complementary figure to Fig 1C to show what female eyes look like in each stock. Note that there is no image of the top "suppressor" or "enhancer" as the repurposing screen was done primarily in males.
(PDF)

S3 Fig Representative images of drug-treated flies. This includes both male and female flies and is complementary to each drug graph displayed here.
(PDF)

S4 Fig Representative images of genetically manipulated flies. This includes both male and females flies and is complementary to S3 Table.
(PDF)

## References

[1] Haendel M, Vasilevsky N, Unni D, Bologa C, Harris N, Rehm H, et al. How many rare diseases are there? Nat Rev Drug Discov. 2020 Feb 1;19(2):77–8. doi: 10.1038/d41573-019-00180-y 32020066 PMC7771654

[2] Ferreira CR. The burden of rare diseases. Am J Med Genet Part A. 2019 Jun 1;179(6):885–92. doi: 10.1002/ajmg.a.61124 30883013

[3] Pushpakom S, Iorio F, Eyers PA, Escott KJ, Hopper S, Wells A, et al. Drug repurposing: progress, challenges and recommendations. Nat Rev Drug Discov. 2018 Oct 12;18(1):41–58. doi: 10.1038/nrd.2018.168 30310233

[4] Meadows WA, Hollowell BD. “Off-label” drug use: an FDA regulatory term, not a negative implication of its medical use. Int J Impot Res. 2008 Mar;20(2):135–44. doi: 10.1038/sj.ijir.3901619 18004389

[5] Bellera CL, Di Ianni ME, Sbaraglini ML, Bruno-Blanch LE, Talevi A, Castro EA. Knowledge-Based Drug Repurposing: A Rational Approach Towards the Identification of Novel Medical Applications of Known Drugs. Front Comput Chem Vol 1 Comput Appl Drug Des Biomol Syst. 2015 Jan 1;44–81.

[6] Wu X, Rush JS, Karaoglu D, Krasnewich D, Lubinsky MS, Waechter CJ, et al. Deficiency of UDP-GlcNac:dolichol phosphate N-acetylglucosamine-1 phosphate transferase (DPAGT1) causes a novel congenital disorder of glycosylation type Ij. Hum Mutat. 2003;22(2):144–50. doi: 10.1002/humu.10239 12872255

[7] Péanne R, de Lonlay P, Foulquier F, Kornak U, Lefeber DJ, Morava E, et al. Congenital disorders of glycosylation (CDG): Quo vadis? Eur J Med Genet. 2018 Nov 1;61(11):643–63. doi: 10.1016/j.ejmg.2017.10.012 29079546

[8] Francisco R, Marques-da-Silva D, Brasil S, Pascoal C, dos Reis Ferreira V, Morava E, et al. The challenge of CDG diagnosis. Mol Genet Metab. 2019 Jan 1;126(1):1–5. doi: 10.1016/j.ymgme.2018.11.003 30454869

[9] Ng BG, Freeze HH, Himmelreich N, Blau N, Ferreira CR. Clinical and biochemical footprints of congenital disorders of glycosylation: Proposed nosology. Mol Genet Metab. 2024 May;142(1):108476. doi: 10.1016/j.ymgme.2024.108476 38653092 PMC11251693

[10] Reily C, Stewart TJ, Renfrow MB, Novak J. Glycosylation in health and disease. Nat Rev Nephrol. 2019 Jun 1;15(6):346–66. doi: 10.1038/s41581-019-0129-4 30858582 PMC6590709

[11] Flynn RA, Pedram K, Malaker SA, Batista PJ, Smith BAH, Johnson AG, et al. Small RNAs are modified with N-glycans and displayed on the surface of living cells. Cell. 2021 Jun 10;184(12):3109–3124.e22. doi: 10.1016/j.cell.2021.04.023 34004145 PMC9097497

[12] Lowenthal MS, Davis KS, Formolo T, Kilpatrick LE, Phinney KW. Identification of Novel N-Glycosylation Sites at Noncanonical Protein Consensus Motifs. J Proteome Res. 2016 Jul 1;15(7):2087–101. doi: 10.1021/acs.jproteome.5b00733 27246700 PMC5100817

[13] Yuste-Checa P, Vega AI, Martín-Higueras C, Medrano C, Gámez A, Desviat LR, et al. DPAGT1-CDG: Functional analysis of disease-causing pathogenic mutations and role of endoplasmic reticulum stress. Lewin AS, editor. PLoS One. 2017 Jun 29;12(6):e0179456. doi: 10.1371/journal.pone.0179456 28662078 PMC5491010

[14] Ng BG, Underhill HR, Palm L, Bengtson P, Rozet JM, Gerber S, et al. DPAGT1 Deficiency with Encephalopathy (DPAGT1-CDG): Clinical and Genetic Description of 11 New Patients. JIMD Rep. 2019;44:85–92. doi: 10.1007/8904_2018_128 30117111 PMC6323016

[15] Belaya K, Finlayson S, Cossins J, Liu WW, Maxwell S, Palace J, et al. Identification of *DPAGT1* as a new gene in which mutations cause a congenital myasthenic syndrome. Ann N Y Acad Sci. 2012 Dec 20;1275(1):29–35. 23278575 10.1111/j.1749-6632.2012.06790.x PMC6044425

[16] Brande L Vanden, Bauché S, Pérez-Guàrdia L, Sternberg D, Seferian AM, Malfatti E, et al. Pathogenic *DPAGT1* variants in limb-girdle congenital myasthenic syndrome (LG-CMS) associated with tubular aggregates and ORAI1 hypoglycosylation. Neuropathol Appl Neurobiol. 2023 Dec 20;e12952. 10.1111/nan.12952 38124360

[17] Selcen D, Shen XM, Brengman J, Li Y, Stans AA, Wieben E, et al. DPAGT1 myasthenia and myopathy: Genetic, phenotypic, and expression studies. Neurology. 2014 May 20;82(20):1822. doi: 10.1212/WNL.0000000000000435 24759841 PMC4035711

[18] Jaeken J, Lefeber D, Matthijs G. Clinical utility gene card for: DPAGT1 defective congenital disorder of glycosylation. Eur J Hum Genet. 2015 Dec 1;23(12):e1–3. doi: 10.1038/ejhg.2015.177 26242989 PMC4795212

[19] Mahesan A, Kamila G, Tiwari R, Das S, Sharma MC, Jauhari P, et al. Congenital Myasthenia Syndrome Due to a Novel DPAGT1 Gene Mutation—An Error of Glycosylation Masquerading as a Congenital Myopathy. Neurol India. 2024 Jan 1;72(1):175–7. doi: 10.4103/neurol-india.Neurol-India-D-23-00582 38443029

[20] Iqbal Z, Shahzad M, Vissers LELM, Van Scherpenzeel M, Gilissen C, Razzaq A, et al. A compound heterozygous mutation in DPAGT1 results in a congenital disorder of glycosylation with a relatively mild phenotype. Eur J Hum Genet. 2013 Aug;21(8):844–9. doi: 10.1038/ejhg.2012.257 23249953 PMC3722673

[21] Reiter LT, Potocki L, Chien S, Gribskov M, Bier E. A systematic analysis of human disease-associated gene sequences in Drosophila melanogaster. Genome Res. 2001;11(6):1114–25. doi: 10.1101/gr.169101 11381037 PMC311089

[22] Su TT. Drug screening in Drosophila; why, when, and when not? Wiley Interdiscip Rev Dev Biol. 2019 Nov 1;8(6):e346. doi: 10.1002/wdev.346 31056843 PMC6786905

[23] Nall AH, Sehgal A. Small-Molecule Screen in Adult Drosophila Identifies VMAT as a Regulator of Sleep. J Neurosci. 2013 May 8;33(19):8534–40. doi: 10.1523/JNEUROSCI.0253-13.2013 23658190 PMC3677510

[24] Yadav AK, Srikrishna S, Gupta SC. Cancer Drug Development Using Drosophila as an in vivo Tool: From Bedside to Bench and Back. Trends Pharmacol Sci. 2016 Sep 1;37(9):789–806. doi: 10.1016/j.tips.2016.05.010 27298020

[25] Hope KA, Berman AR, Peterson RT, Chow CY. An in vivo drug repurposing screen and transcriptional analyses reveals the serotonin pathway and GSK3 as major therapeutic targets for NGLY1 deficiency. Girirajan S, editor. PLOS Genet. 2022 Jun 2;18(6):e1010228. doi: 10.1371/journal.pgen.1010228 35653343 PMC9162339

[26] Iyer S, Sam FS, DiPrimio N, Preston G, Verheijen J, Murthy K, et al. Repurposing the aldose reductase inhibitor and diabetic neuropathy drug epalrestat for the congenital disorder of glycosylation PMM2-CDG. Dis Model Mech. 2019;12(11). doi: 10.1242/dmm.040584 31636082 PMC6899038

[27] Ligezka AN, Radenkovic S, Saraswat M, Garapati K, Ranatunga W, Krzysciak W, et al. Sorbitol Is a Severity Biomarker for PMM2-CDG with Therapeutic Implications. Ann Neurol. 2021 Dec 1;90(6):887–900. doi: 10.1002/ana.26245 34652821 PMC8820356

[28] Dalton HM, Viswanatha R Jr. RB, Zuno JS, Berman AR, Rushforth R, et al. A genome-wide CRISPR screen identifies DPM1 as a modifier of DPAGT1 deficiency and ER stress. PLOS Genet. 2022 Sep 27;18(9):e1010430. doi: 10.1371/journal.pgen.1010430 36166480 PMC9543880

[29] Thomas BJ, Wassarman DA. A fly’s eye view of biology. Trends Genet. 1999 May 1;15(5):184–90. doi: 10.1016/s0168-9525(99)01720-5 10322485

[30] Pletcher RC, Hardman SL, Intagliata SF, Lawson RL, Page A, Tennessen JM. A Genetic Screen Using the Drosophila melanogaster TRiP RNAi Collection To Identify Metabolic Enzymes Required for Eye Development. G3 (Bethesda). 2019 Jul 1;9(7):2061–70. doi: 10.1534/g3.119.400193 31036678 PMC6643872

[31] Iyer J, Wang Q, Le T, Pizzo L, Grönke S, Ambegaokar SS, et al. Quantitative assessment of eye phenotypes for functional genetic studies using Drosophila melanogaster. G3 Genes, Genomes, Genet. 2016 May 1;6(5):1427–37. doi: 10.1534/g3.116.027060 26994292 PMC4856093

[32] Cutler T, Sarkar A, Moran M, Steffensmeier A, Puli OR, Mancini G, et al. Drosophila Eye Model to Study Neuroprotective Role of CREB Binding Protein (CBP) in Alzheimer’s Disease. Pandey U, editor. PLoS One. 2015 Sep 14;10(9):e0137691. doi: 10.1371/journal.pone.0137691 26367392 PMC4569556

[33] Brand AH, Perrimon N. Targeted gene expression as a means of altering cell fates and generating dominant phenotypes. Development. 1993;118(2):401–15. doi: 10.1242/dev.118.2.401 8223268

[34] Weasner BM, Weasner BP, Neuman SD, Bashirullah A, Kumar JP. Retinal Expression of the Drosophila eyes absent Gene Is Controlled by Several Cooperatively Acting Cis-regulatory Elements. PLOS Genet. 2016 Dec 1;12(12):e1006462. doi: 10.1371/journal.pgen.1006462 27930646 PMC5145141

[35] Dong YY, Wang H, Pike ACW, Cochrane SA, Hamedzadeh S, Wyszyński FJ, et al. Structures of DPAGT1 Explain Glycosylation Disease Mechanisms and Advance TB Antibiotic Design. Cell. 2018 Nov 1;175(4):1045–1058.e16. doi: 10.1016/j.cell.2018.10.037 30388443 PMC6218659

[36] Levine BD, Cagan RL. Drosophila Lung Cancer Models Identify Trametinib Plus Statin as Candidate Therapeutic. Cell Rep. 2016 Feb 16;14(6):1477. doi: 10.1016/j.celrep.2015.12.105 26832408 PMC4904304

[37] Chang S, Bray SM, Li Z, Zarnescu DC, He C, Jin P, et al. Identification of small molecules rescuing fragile X syndrome phenotypes in Drosophila. Nat Chem Biol. 2008 Mar 9;4(4):256–63. doi: 10.1038/nchembio.78 18327252

[38] Gehle VM, Walcott EC, Nishizaki T, Sumikawa K. N-Glycosylation at the conserved sites ensures the expression of properly folded functional ACh receptors. Mol Brain Res. 1997 May;45(2):219–29. doi: 10.1016/s0169-328x(96)00256-2 9149096

[39] Bruning TA, Chang PC, Hendriks MGC, Vermeij P, Pfaffendorf M, Van Zwieten PA. In vivo characterization of muscarinic receptor subtypes that mediate vasodilatation in patients with essential hypertension. Hypertension. 1995;26(1):70–7. doi: 10.1161/01.hyp.26.1.70 7607735

[40] Bodur E, Çokuǧraş AN, Tezcan EF. Inhibition effects of benactyzine and drofenine on human serum butyrylcholinesterase. Arch Biochem Biophys. 2001 Feb 1;386(1):25–9. doi: 10.1006/abbi.2000.2188 11360997

[41] Hindmarsh J, Woods E, Lee M, Pickard J. Administering Neostigmine as a Subcutaneous Infusion: A Case Report of a Patient Dying With Myasthenia Gravis. J Palliat Care. 2020 Apr 1;35(2):78–81. doi: 10.1177/0825859719869353 31411109

[42] Finlayson S, Palace J, Belaya K, Walls TJ, Norwood F, Burke G, et al. Clinical features of congenital myasthenic syndrome due to mutations in DPAGT1. J Neurol Neurosurg Psychiatry. 2013;84(10):1119. doi: 10.1136/jnnp-2012-304716 23447650 PMC6044426

[43] Gai Y, Liu Z, Cervantes-Sandoval I, Davis RL. Drosophila SLC22A Transporter Is a Memory Suppressor Gene that Influences Cholinergic Neurotransmission to the Mushroom Bodies. Neuron. 2016 May 4;90(3):581–95. doi: 10.1016/j.neuron.2016.03.017 27146270 PMC4894652

[44] Yamamoto S, Seto ES. Dopamine dynamics and signaling in Drosophila: An overview of genes, drugs and behavioral paradigms. Exp Anim. 2014;63(2):107–19. doi: 10.1538/expanim.63.107 24770636 PMC4160991

[45] Karam CS, Jones SK, Javitch JA. Come Fly with Me: An overview of dopamine receptors in Drosophila melanogaster. Basic Clin Pharmacol Toxicol. 2020 Jun 1;126(S6):56–65. doi: 10.1111/bcpt.13277 31219669 PMC6923619

[46] Eisenegger C, Naef M, Linssen A, Clark L, Gandamaneni PK, Müller U, et al. Role of Dopamine D2 Receptors in Human Reinforcement Learning. Neuropsychopharmacology. 2014 Apr 9;39(10):2366–75. doi: 10.1038/npp.2014.84 24713613 PMC4138746

[47] Li P, L. Snyder G, E. Vanover K. Dopamine Targeting Drugs for the Treatment of Schizophrenia: Past, Present and Future. Curr Top Med Chem. 2016 Jun 18;16(29):3385–403. doi: 10.2174/1568026616666160608084834 27291902 PMC5112764

[48] Din L, Preuss C V. Prochlorperazine. xPharm Compr Pharmacol Ref. 2023 Aug 14;1–6.

[49] Friedman BW, Esses D, Solorzano C, Dua N, Greenwald P, Radulescu R, et al. A Randomized Controlled Trial of Prochlorperazine Versus Metoclopramide for Treatment of Acute Migraine. Ann Emerg Med. 2008 Oct 1;52(4):399–406. doi: 10.1016/j.annemergmed.2007.09.027 18006188

[50] Jenkins G. Review of Dopamine Antagonists for Nausea and Vomiting in Palliative Care Patients. J Pain Palliat Care Pharmacother. 2023; doi: 10.1080/15360288.2023.2268065 37843383

[51] Seeman P, Corbettand R, Van Tol HHM. Atypical neuroleptics have low affinity for dopamine D2 receptors or are selective for D4 receptors. Neuropsychopharmacology. 1997 Feb 1;16(2):93–110. doi: 10.1016/S0893-133X(96)00187-X 9015795

[52] Deng B, Li Q, Liu X, Cao Y, Li B, Qian Y, et al. Chemoconnectomics: Mapping Chemical Transmission in Drosophila. Neuron. 2019 Mar 6;101(5):876–893.e4. doi: 10.1016/j.neuron.2019.01.045 30799021

[53] Handler A, Graham TGW, Cohn R, Morantte I, Siliciano AF, Zeng J, et al. Distinct Dopamine Receptor Pathways Underlie the Temporal Sensitivity of Associative Learning. Cell. 2019 Jun 27;178(1):60–75.e19. doi: 10.1016/j.cell.2019.05.040 31230716 PMC9012144

[54] Katz JL, Izenwasser S, Terry P. Relationships among dopamine transporter affinities and cocaine-like discriminative-stimulus effects. Psychopharmacology (Berl). 2000;148(1):90–8. doi: 10.1007/s002130050029 10663422

[55] Salvatore MF, Kasanga EA, Kelley DP, Venable KE, McInnis TR, Cantu MA, et al. Modulation of nigral dopamine signaling mitigates parkinsonian signs of aging: evidence from intervention with calorie restriction or inhibition of dopamine uptake. GeroScience. 2023 Feb 1;45(1):45. doi: 10.1007/s11357-022-00583-7 35635679 PMC9886753

[56] Wang CH, Chen II, Chen CH, Tseng YT. Pharmacoepidemiological Research on N-Nitrosodimethylamine-Contaminated Ranitidine Use and Long-Term Cancer Risk: A Population-Based Longitudinal Cohort Study. Int J Environ Res Public Health. 2022 Sep 30;19(19):12469. doi: 10.3390/ijerph191912469 36231768 PMC9566239

[57] Borycz J, Borycz JA, Loubani M, Meinertzhagen IA. tan and ebony genes regulate a novel pathway for transmitter metabolism at fly photoreceptor terminals. J Neurosci. 2002 Dec 15;22(24):10549–57. doi: 10.1523/JNEUROSCI.22-24-10549.2002 12486147 PMC6758454

[58] Wright TRF. The genetics of biogenic amine metabolism, sclerotization, and melanization in drosophila melanogaster. Adv Genet. 1987 Jan 1;24(C):127–222. 3124532

[59] Koski SK, Leino S, Panula P, Rannanpää S, Salminen O. Genetic lack of histamine upregulates dopamine neurotransmission and alters rotational behavior but not levodopa-induced dyskinesia in a mouse model of Parkinson’s disease. Neurosci Lett. 2020 Jun 11;729. doi: 10.1016/j.neulet.2020.134932 32224226

[60] Perz JB, Ho AD. Histamine dihydrochloride for the treatment of acute myeloid leukemia, malignant melanoma and renal cell carcinoma. Futur Oncol. 2008 Apr;4(2):169–77. doi: 10.2217/14796694.4.2.169 18407731

[61] Delpire E, Gagnon KB. Na+-K+-2Cl- cotransporter (NKCC) physiological function in nonpolarized cells and transporting epithelia. Compr Physiol. 2018 Apr 1;8(2):871–901. doi: 10.1002/cphy.c170018 29687903

[62] Lykke K, Töllner K, Feit PW, Erker T, MacAulay N, Löscher W. The search for NKCC1-selective drugs for the treatment of epilepsy: Structure–function relationship of bumetanide and various bumetanide derivatives in inhibiting the human cation-chloride cotransporter NKCC1A. Epilepsy Behav. 2016 Jun 1;59:42–9. doi: 10.1016/j.yebeh.2016.03.021 27088517

[63] Kharod SC, Kang SK, Kadam SD. Off-label use of bumetanide for brain disorders: An overview. Front Neurosci. 2019;13(APR). doi: 10.3389/fnins.2019.00310 31068771 PMC6491514

[64] Kahle KT, Staley KJ. The bumetanide-sensitive Na-K-2Cl cotransporter NKCC1 as a potential target of a novel mechanism-based treatment strategy for neonatal seizures. Neurosurg Focus. 2008;25(3). doi: 10.3171/FOC/2008/25/9/E22 18759624

[65] Zhao Y, Roy K, Vidossich P, Cancedda L, De Vivo M, Forbush B, et al. Structural basis for inhibition of the Cation-chloride cotransporter NKCC1 by the diuretic drug bumetanide. Nat Commun. 2022 Dec 1;13(1):1–12. 35585053 10.1038/s41467-022-30407-3 PMC9117670

[66] Talsness DM, Owings KG, Coelho E, Mercenne G, Pleinis JM, Partha R, et al. A Drosophila screen identifies NKCC1 as a modifier of NGLY1 deficiency. Elife. 2020 Dec 1;9:1–22. doi: 10.7554/eLife.57831 33315011 PMC7758059

[67] Leiserson WM, Forbush B, Keshishian H. Drosophila glia use a conserved cotransporter mechanism to regulate extracellular volume. Glia. 2011 Feb;59(2):320–32. doi: 10.1002/glia.21103 21125654 PMC3005002

[68] Turini ME, DuBois RN. Cyclooxygenase-2: A therapeutic target. Annu Rev Med. 2002;53:35–57. doi: 10.1146/annurev.med.53.082901.103952 11818462

[69] Simmons DL, Botting RM, Hla T. Cyclooxygenase isozymes: The biology of prostaglandin synthesis and inhibition. Pharmacol Rev. 2004 Sep;56(3):387–437. doi: 10.1124/pr.56.3.3 15317910

[70] Yang H, Chen C. Cyclooxygenase-2 in Synaptic Signaling. Curr Pharm Des. 2008 May 22;14(14):1443–51. doi: 10.2174/138161208784480144 18537667 PMC2561288

[71] Duran X, Sánchez S, Vilahur G, Badimon L. Protective effects of triflusal on secondary thrombus growth and vascular cyclooxygenase-2. J Thromb Haemost. 2008 Aug;6(8):1385–92. doi: 10.1111/j.1538-7836.2008.03036.x 18503633

[72] Brodie BB, Axelrod J. The fate of antipyrine in man. J Pharmacol Exp Ther. 1950 Jan;98(1):97–104. 15403625

[73] Zhou Y, Boudreau DM, Freedman AN. Trends in the use of aspirin and nonsteroidal anti-inflammatory drugs in the general U.S. population. Pharmacoepidemiol Drug Saf. 2014 Jan;23(1):43–50. doi: 10.1002/pds.3463 23723142

[74] Stiller CO, Hjemdahl P. Lessons from 20 years with COX-2 inhibitors: Importance of dose–response considerations and fair play in comparative trials. J Intern Med. 2022 Oct 1;292(4):557–74. doi: 10.1111/joim.13505 35585779

[75] Huang K, Masuda A, Chen G, Bushra S, Kamon M, Araki T, et al. Inhibition of cyclooxygenase-1 by nonsteroidal anti-inflammatory drugs demethylates MeR2 enhancer and promotes Mbnl1 transcription in myogenic cells. Sci Rep. 2020 Dec 1;10(1). doi: 10.1038/s41598-020-59517-y 32054946 PMC7018979

[76] Malskat WS, Knulst AC, Bruijnzeel-Koomen CA, Röckmann H. Tolerance to alternative cyclooxygenase-2 inhibitors in nonsteroidal anti-inflammatory drug hypersensitive patients. Clin Transl Allergy. 2013 Jun 24;3(1):1–7. 23799898 10.1186/2045-7022-3-20 PMC3704733

[77] Weberschock TB, Müller SM, Boehncke S, Boehncke WH. Tolerance to coxibs in patients with intolerance to non-steroidal anti-inflammatory drugs (NSAIDs): A systematic structured review of the literature. Arch Dermatol Res. 2007 Jul;299(4):169–75. doi: 10.1007/s00403-007-0757-6 17492455 PMC1910889

[78] LiverTox: Clinical and Research Information on Drug-Induced Liver Injury [Internet]. 2012 [cited 2024 May 28]. Tolmetin. Available from: https://www.ncbi.nlm.nih.gov/books/NBK547852/. 31643176

[79] Tootle TL, Spradling AC. Drosophila Pxt: A cyclooxygenase-like facilitator of follicle maturation. Development. 2008 Mar;135(5):839–47. doi: 10.1242/dev.017590 18216169 PMC2818214

[80] Spracklen AJ, Kelpsch DJ, Chen X, Spracklen CN, Tootle TL. Prostaglandins temporally regulate cytoplasmic actin bundle formation during Drosophila oogenesis. Mol Biol Cell. 2014 Feb 1;25(3):397–411. doi: 10.1091/mbc.E13-07-0366 24284900 PMC3907279

[81] Mehndiratta MM, Pandey S, Kuntzer T. Acetylcholinesterase inhibitor treatment for myasthenia gravis. Cochrane Database Syst Rev. 2014 Oct 13;2014(10). doi: 10.1002/14651858.CD006986.pub3 25310725 PMC7390275

[82] Jan LY, Jan YN. L-glutamate as an excitatory transmitter at the Drosophila larval neuromuscular junction. J Physiol. 1976 Oct 1;262(1):215–36. doi: 10.1113/jphysiol.1976.sp011593 186587 PMC1307638

[83] Pyakurel P, Shin M, Venton BJ. Nicotinic acetylcholine receptor (nAChR) mediated dopamine release in larval Drosophila melanogaster. Neurochem Int. 2018 Mar 1;114:33–41. doi: 10.1016/j.neuint.2017.12.012 29305920 PMC5835409

[84] Koranda JL, Cone JJ, McGehee DS, Roitman MF, Beeler JA, Zhuang X. Nicotinic receptors regulate the dynamic range of dopamine release in vivo. J Neurophysiol. 2014 Jan 1;111(1):103–11. doi: 10.1152/jn.00269.2013 24089398 PMC3921376

[85] Zhou FM, Liang Y, Dani JA. Endogenous nicotinic cholinergic activity regulates dopamine release in the striatum. Nat Neurosci. 2001 Nov 19;4(12):1224–9. doi: 10.1038/nn769 11713470

[86] Brimijoin S, Koenigsberger C. Cholinesterases in neural development: New findings and toxicologic implications. Environ Health Perspect. 1999;107(SUPPL. 1):59–64. doi: 10.1289/ehp.99107s159 10229707 PMC1566370

[87] Hankin MH, Hoover F, Goldman D. Cues intrinsic to the retina induce nAchR gene expression during development. J Neurobiol. 1993;24(8):1099–110. doi: 10.1002/neu.480240808 8409970

[88] Hohmann CF, Brooks AR, Coyle JT. Neonatal lesions of the basal forebrain cholinergic neurons result in abnormal cortical development. Dev Brain Res. 1988 Aug 1;42(2):253–64. doi: 10.1016/0165-3806(88)90244-1 3219585

[89] Lauder JM, Schambra UB. Morphogenetic roles of acetylcholine. Environ Health Perspect. 1999;107(SUPPL. 1):65–9. doi: 10.1289/ehp.99107s165 10229708 PMC1566361

[90] Lecomte MJ, Bertolus C, Ramanantsoa N, Saurini F, Callebert J, Sénamaud-Beaufort C, et al. Acetylcholine Modulates the Hormones of the Growth Hormone/Insulinlike Growth Factor-1 Axis During Development in Mice. Endocrinology. 2018 Apr 1;159(4):1844–59. doi: 10.1210/en.2017-03175 29509880

[91] Cisterna BA, Vargas AA, Puebla C, Fernández P, Escamilla R, Lagos CF, et al. Active acetylcholine receptors prevent the atrophy of skeletal muscles and favor reinnervation. Nat Commun. 2020 Dec 1;11(1):1–13. 32103010 10.1038/s41467-019-14063-8 PMC7044284

[92] Özsoy Ö, Cinleti T, Günay Ç, Sarlkaya Uzan G, Yeşilmen MC, Lochmüller H, et al. DPAGT1-CDG: Report of Two New Pediatric Patients and Brief Review of the Literature. Mol Syndromol. 2023 Aug 1;14(4):322–30. doi: 10.1159/000529494 37766827 PMC10521235

[93] Boczek T, Cameron EG, Yu W, Xia X, Shah SH, Chabeco BC, et al. Regulation of neuronal survival and axon growth by a perinuclear cAMP compartment. J Neurosci. 2019 Jul 10;39(28):5466–80. doi: 10.1523/JNEUROSCI.2752-18.2019 31097623 PMC6616289

[94] Suzuki S, Yokoyama U, Abe T, Kiyonari H, Yamashita N, Kato Y, et al. Differential roles of Epac in regulating cell death in neuronal and myocardial cells. J Biol Chem. 2010 Jul 30;285(31):24248–59. doi: 10.1074/jbc.M109.094581 20516079 PMC2911347

[95] Dewey RB. Management of motor complications in Parkinson’s disease. Neurology. 2004 Mar 23;62(6 Suppl 4):S3–7. doi: 10.1212/wnl.62.6_suppl_4.s3 15037664

[96] Channer B, Matt SM, Nickoloff-Bybel EA, Pappa V, Agarwal Y, Wickman J, et al. Dopamine, Immunity, and Disease. Pharmacol Rev. 2023 Jan 1;75(1):62–158. doi: 10.1124/pharmrev.122.000618 36757901 PMC9832385

[97] Basiri K, Belaya K, Liu WW, Maxwell S, Sedghi M, Beeson D. Clinical features in a large Iranian family with a limb-girdle congenital myasthenic syndrome due to a mutation in DPAGT1. Neuromuscul Disord. 2013 Jun;23(6):469–72. doi: 10.1016/j.nmd.2013.03.003 23591138 PMC3746154

[98] Tuplin EW, Holahan MR. Aripiprazole, A Drug that Displays Partial Agonism and Functional Selectivity. Curr Neuropharmacol. 2017 Apr 17;15(8):1192. doi: 10.2174/1570159X15666170413115754 28412910 PMC5725548

[99] Iyer S, Mast JD, Tsang H, Rodriguez TP, DiPrimio N, Prangley M, et al. Drug screens of NGLY1 deficiency in worm and fly models reveal catecholamine, NRF2 and anti-inflammatory-pathway activation as potential clinical approaches. DMM Dis Model Mech. 2019 Nov 1;12(11). doi: 10.1242/dmm.040576 31615832 PMC6899034

[100] Consortium TU, Bateman A, Martin MJ, Orchard S, Magrane M, Agivetova R, et al. UniProt: the universal protein knowledgebase in 2021. Nucleic Acids Res. 2021 Jan 8;49(D1):D480–9. doi: 10.1093/nar/gkaa1100 33237286 PMC7778908

[101] Singh R, Almutairi MM, Pacheco-Andrade R, Almiahuob MYM, Di Fulvio M. Impact of Hybrid and Complex N-Glycans on Cell Surface Targeting of the Endogenous Chloride Cotransporter Slc12a2. Int J Cell Biol. 2015;2015. doi: 10.1155/2015/505294 26351455 PMC4553341

[102] Paredes A, Plata C, Rivera M, Moreno E, Vázquez N, Muñoz-Clares R, et al. Activity of the renal Na+-K+-2Cl- cotransporter is reduced by mutagenesis of N-glycosylation sites: Role for protein surface charge in Cl- transport. Am J Physiol—Ren Physiol. 2006;290(5). 10.1152/ajprenal.00071.2005 16291577

[103] Löscher W, Kaila K. CNS pharmacology of NKCC1 inhibitors. Neuropharmacology. 2022 Mar 1;205. doi: 10.1016/j.neuropharm.2021.108910 34883135

[104] Sivakumaran S, Maguire J. Bumetanide reduces seizure progression and the development of pharmacoresistant status epilepticus. Epilepsia. 2016 Feb 1;57(2):222–32. doi: 10.1111/epi.13270 26659482 PMC5487491

[105] Soul JS, Bergin AM, Stopp C, Hayes B, Singh A, Fortuno CR, et al. A Pilot Randomized, Controlled, Double-Blind Trial of Bumetanide to Treat Neonatal Seizures. Ann Neurol. 2021 Feb 1;89(2):327–40. doi: 10.1002/ana.25959 33201535 PMC8122513

[106] Fuentes J, Parellada M, Georgoula C, Oliveira G, Marret S, Crutel V, et al. Bumetanide oral solution for the treatment of children and adolescents with autism spectrum disorder: Results from two randomized phase III studies. Autism Res. 2023 Oct 1;16(10):2021–34. doi: 10.1002/aur.3005 37794745

[107] van Andel DM, Sprengers JJ, Königs M, de Jonge M V., Bruining H. Effects of Bumetanide on Neurocognitive Functioning in Children with Autism Spectrum Disorder: Secondary Analysis of a Randomized Placebo-Controlled Trial. J Autism Dev Disord. 2023 Jan 10;54(3):894–904. doi: 10.1007/s10803-022-05841-3 36626004 PMC10907457

[108] Kim S, Chen J, Cheng T, Gindulyte A, He J, He S, et al. PubChem 2023 update. Nucleic Acids Res. 2023 Jan 6;51(D1):D1373–80. doi: 10.1093/nar/gkac956 36305812 PMC9825602

[109] Brogden RN, Heel RC, Speight TM, Avery GS. Tolmetin: A Review of its Pharmacological Properties and Therapeutic Efficacy in Rheumatic Diseases. Drugs. 1978 Nov 9;15(6):429–50. doi: 10.2165/00003495-197815060-00002 350558

[110] Adam D, Federspil P, Lukes M, Petrowicz O. Therapeutic properties and tolerance of procaine and phenazone containing ear drops in infants and very young children. Arzneimittel-Forschung/Drug Res. 2009;59(10):504–12. doi: 10.1055/s-0031-1296434 19998578

[111] Mahmud S, Rosen N. History of NSAID Use in the Treatment of Headaches Pre and Post-industrial Revolution in the United States: the Rise and Fall of Antipyrine, Salicylic Acid, and Acetanilide. Curr Pain Headache Rep. 2019 Jan 1;23(1). doi: 10.1007/s11916-019-0744-6 30673879

[112] Nadeem M, Maqdoom M. Evaluation of anticonvulsant effect of celecoxib, a selective cyclooxygenase-2 inhibitor in experimentally induced convulsions in albino rats. Int J Basic Clin Pharmacol. 2016;5(4):1466–70.

[113] Chauhan G, Roy K, Kumar G, Kumari P, Alam S, Kishore K, et al. Distinct influence of COX-1 and COX-2 on neuroinflammatory response and associated cognitive deficits during high altitude hypoxia. Neuropharmacology. 2019 Mar 1;146:138–48. doi: 10.1016/j.neuropharm.2018.11.026 30476507

[114] Leucht S, Crippa A, Siafis S, Patel MX, Orsini N, Davis JM. Dose-response meta-analysis of antipsychotic drugs for acute schizophrenia. Am J Psychiatry. 2020 Apr 1;177(4):342–53. doi: 10.1176/appi.ajp.2019.19010034 31838873

[115] Asahina K. Sex differences in Drosophila behavior: qualitative and quantitative dimorphism. Curr Opin Physiol. 2018 Dec 1;6:35–45. doi: 10.1016/j.cophys.2018.04.004 30386833 PMC6205217

[116] Shingleton AW, Vea IM. Sex-specific regulation of development, growth and metabolism. Semin Cell Dev Biol. 2023 Mar 30;138:117–27. doi: 10.1016/j.semcdb.2022.04.017 35469676

[117] Belmonte RL, Corbally MK, Duneau DF, Regan JC. Sexual Dimorphisms in Innate Immunity and Responses to Infection in Drosophila melanogaster. Front Immunol. 2020 Jan 31;10. doi: 10.3389/fimmu.2019.03075 32076419 PMC7006818

[118] Regan JC, Lu YX, Ureña E, Meilenbrock RL, Catterson JH, Kißler D, et al. Sexual identity of enterocytes regulates autophagy to determine intestinal health, lifespan and responses to rapamycin. Nat Aging. 2022 Dec 1;2(12):1145–58. doi: 10.1038/s43587-022-00308-7 37118538 PMC10154239

[119] Khoury GA, Baliban RC, Floudas CA. Proteome-wide post-translational modification statistics: frequency analysis and curation of the swiss-prot database. Sci Rep. 2011 Sep 13;1(1):1–5. doi: 10.1038/srep00090 22034591 PMC3201773

[120] Xu C, Ng DTW. Glycosylation-directed quality control of protein folding. Nat Rev Mol Cell Biol. 2015 Oct 14;16(12):742–52. doi: 10.1038/nrm4073 26465718

[121] Velan B, Kronman C, Ordentlich A, Flashner Y, Leitner M, Cohen S, et al. N-glycosylation of human acetylcholinesterase: Effects on activity, stability and biosynthesis. Biochem J. 1993 Dec 12;296(3):649–56. doi: 10.1042/bj2960649 8280063 PMC1137746

[122] Missale C, Russel Nash S, Robinson SW, Jaber M, Caron MG. Dopamine receptors: From structure to function. Physiol Rev. 1998;78(1):189–225. doi: 10.1152/physrev.1998.78.1.189 9457173

[123] Min C, Zheng M, Zhang X, Guo S, Kwon KJ, Shin CY, et al. N-linked Glycosylation on the N-terminus of the dopamine D2 and D3 receptors determines receptor association with specific microdomains in the plasma membrane. Biochim Biophys Acta—Mol Cell Res. 2015 Jan 1;1853(1):41–51. doi: 10.1016/j.bbamcr.2014.09.024 25289757

[124] Li L Bin, Chen N, Ramamoorthy S, Chi L, Cui XN, Wang LC, et al. The role of N-glycosylation in function and surface trafficking of the human dopamine transporter. J Biol Chem. 2004 May 14;279(20):21012–20. doi: 10.1074/jbc.M311972200 15024013

[125] Chandrasekharan N V., Simmons DL. The cyclooxygenases. Genome Biol. 2004;5(9):241. doi: 10.1186/gb-2004-5-9-241 15345041 PMC522864

[126] Maugars G, Nourizadeh-Lillabadi R, Weltzien FA. New Insights Into the Evolutionary History of Melatonin Receptors in Vertebrates, With Particular Focus on Teleosts. Front Endocrinol (Lausanne). 2020 Sep 24;11:538196. doi: 10.3389/fendo.2020.538196 33071966 PMC7541902

[127] Chazot PL, Cik M, Stephenson FA. An investigation into the role of N-glycosylation in the functional expression of a recombinant heteromeric NMDA receptor. Mol Membr Biol. 1995;12(4):331–7. doi: 10.3109/09687689509072435 8747278

[128] Janezic EM, Lauer SML, Williams RG, Chungyoun M, Lee KS, Navaluna E, et al. N-glycosylation of α1D-adrenergic receptor N-terminal domain is required for correct trafficking, function, and biogenesis. Sci Rep. 2020 Dec 1;10(1). 10.1038/s41598-020-64102-4 PMC7190626 32350295

[129] Li X, Zhou M, Huang W, Yang H. N-glycosylation of the β2 adrenergic receptor regulates receptor function by modulating dimerization. FEBS J. 2017 Jul 1;284(13):2004–18. 28467637 10.1111/febs.14098

[130] Dietzl G, Chen D, Schnorrer F, Su KC, Barinova Y, Fellner M, et al. A genome-wide transgenic RNAi library for conditional gene inactivation in Drosophila. Nature. 2007 Jul 12;448(7150):151–6. doi: 10.1038/nature05954 17625558

[131] Chow CY, Kelsey KJP, Wolfner MF, Clark AG. Candidate genetic modifiers of retinitis pigmentosa identified by exploiting natural variation in Drosophila. Hum Mol Genet. 2016 Feb 15;25(4):651–9. doi: 10.1093/hmg/ddv502 26662796 PMC4743685

[132] Neely GG, Hess A, Costigan M, Keene AC, Goulas S, Langeslag M, et al. A Genome-wide Drosophila Screen for Heat Nociception Identifies α2δ3 as an Evolutionarily Conserved Pain Gene. Cell. 2010 Nov 12;143(4):628–38. 21074052 10.1016/j.cell.2010.09.047 PMC3040441

[133] Czech B, Preall JB, McGinn J, Hannon GJ. A Transcriptome-wide RNAi Screen in the Drosophila Ovary Reveals Factors of the Germline piRNA Pathway. Mol Cell. 2013 Jun 6;50(5):749–61. doi: 10.1016/j.molcel.2013.04.007 23665227 PMC3724427

[134] Baumbach J, Hummel P, Bickmeyer I, Kowalczyk KM, Frank M, Knorr K, et al. A Drosophila In Vivo Screen Identifies Store-Operated Calcium Entry as a Key Regulator of Adiposity. Cell Metab. 2014 Feb 4;19(2):331–43. 24506874 10.1016/j.cmet.2013.12.004

