# Supplementary figures and images for: A drug repurposing screen reveals dopamine signaling as a critical pathway underlying potential therapeutics for the rare disease DPAGT1-CDG
Source: PLoS Genet. 2024 Oct 28;20(10):e1011458. doi: 10.1371/journal.pgen.1011458 (PMC11542785; doi:10.1371/journal.pgen.1011458)

**A.***DPAGT1*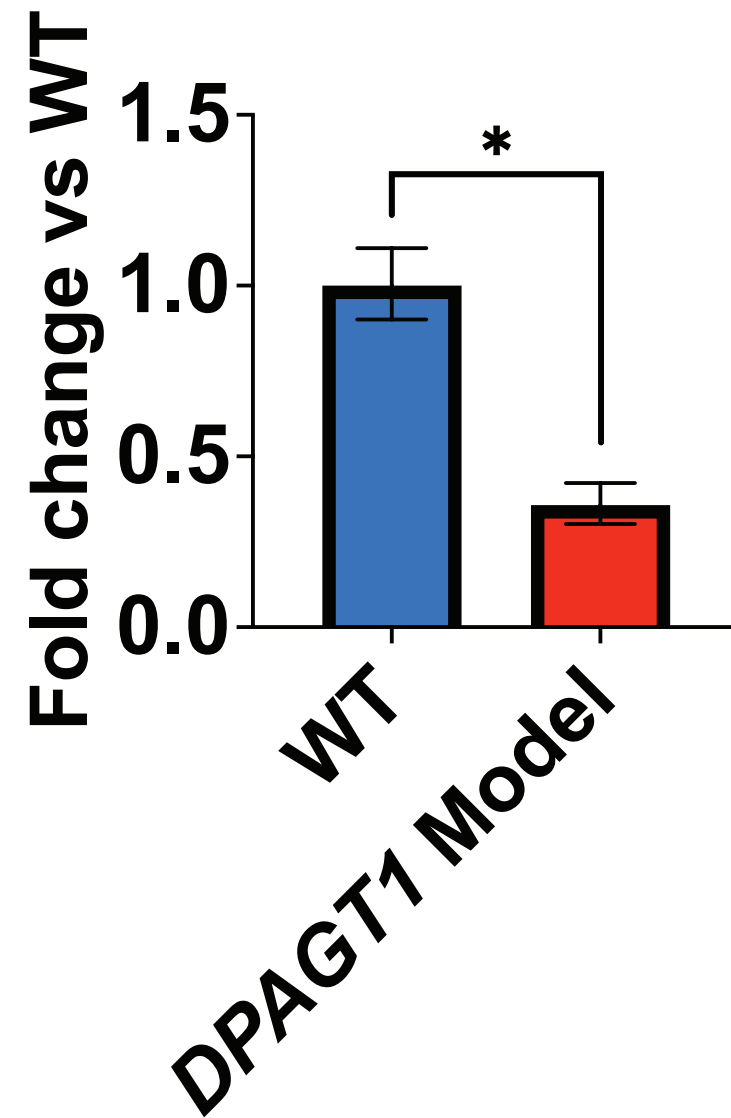**B.**Second *DPAGT1* model (BDSC 51869 stock)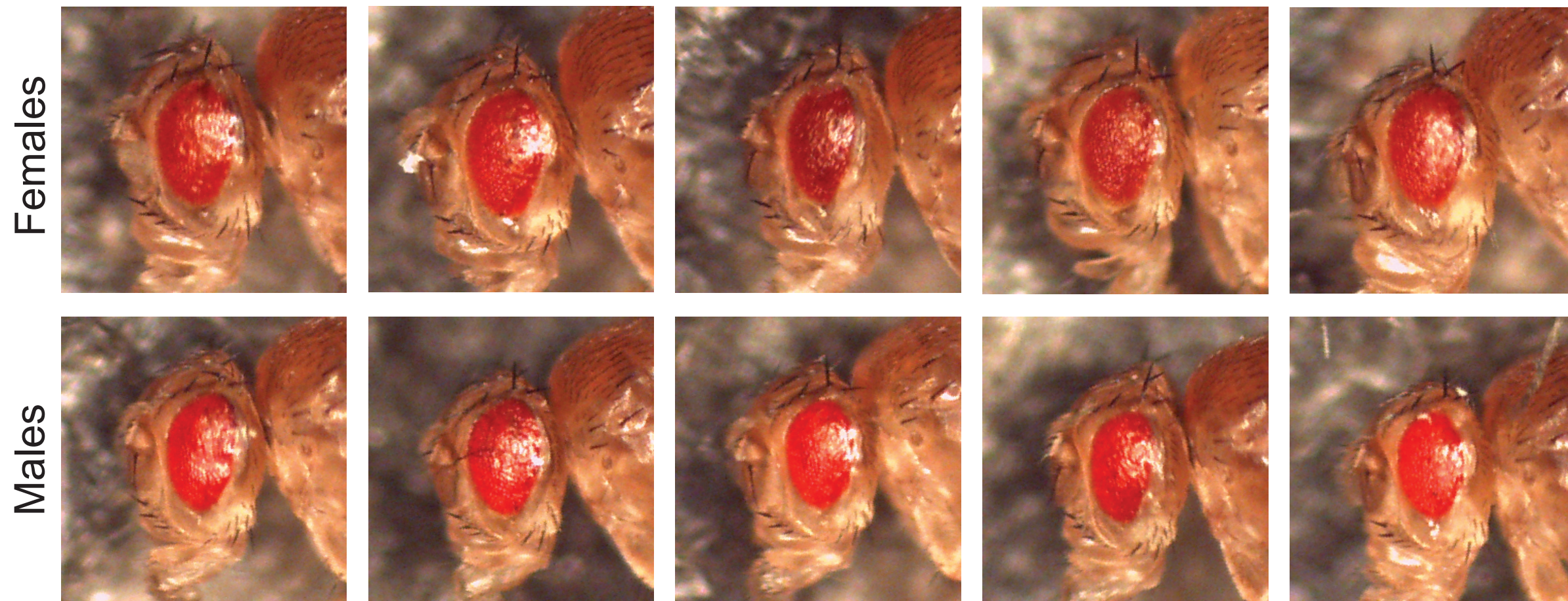

Supplement: S1 Fig — (A) Graph of DPAGT1 knockdown in the DPAGT1 model, * p<0.05, (Student’s t-test). (B) Representative images of a second DPAGT1 knockdown model using the BDSC 51869 stock. (PDF) [file pgen.1011458.s005.pdf]

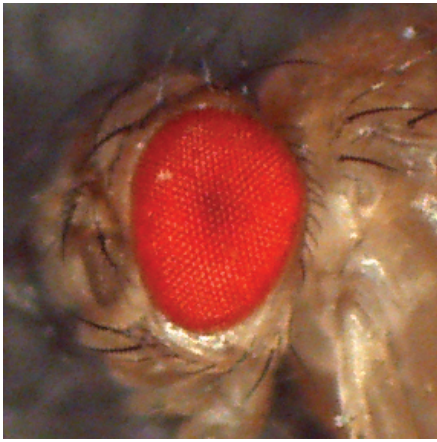

*eya*  
composite-GAL4

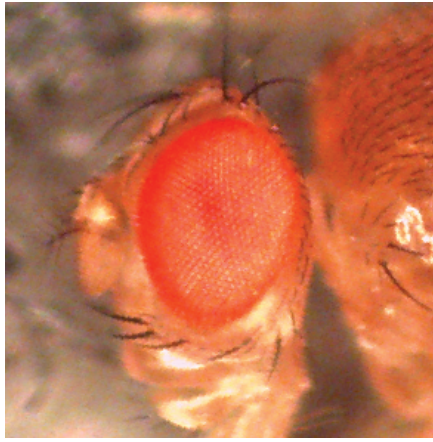

RNAi control  
BDSC 36303

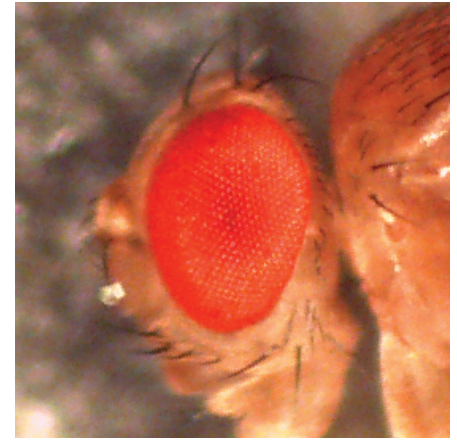

*DPAGT1* RNAi  
BDSC 53264

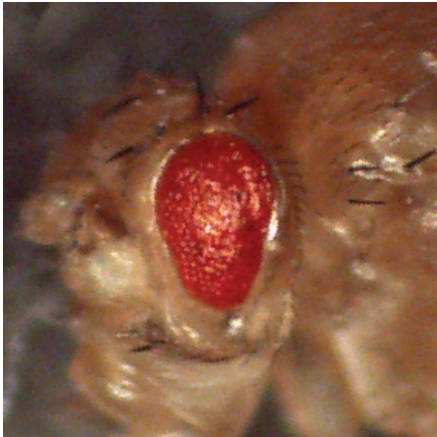

*DPAGT1* Model

Females

Supplement: S2 Fig — This is a complementary figure to Fig 1C to show what female eyes look like in each stock. Note that there is no image of the top "suppressor" or "enhancer" as the repurposing screen was done primarily in males. (PDF) [file pgen.1011458.s006.pdf]
