# Supplementary material for: A drug repurposing screen reveals dopamine signaling as a critical pathway underlying potential therapeutics for the rare disease DPAGT1-CDG
Source: PLoS Genet. 2024 Oct 28;20(10):e1011458. doi: 10.1371/journal.pgen.1011458 (PMC11542785; doi:10.1371/journal.pgen.1011458)
Supplement: S3 Fig — This includes both male and female flies and is complementary to each drug graph displayed here. (PDF) [file pgen.1011458.s007.pdf]

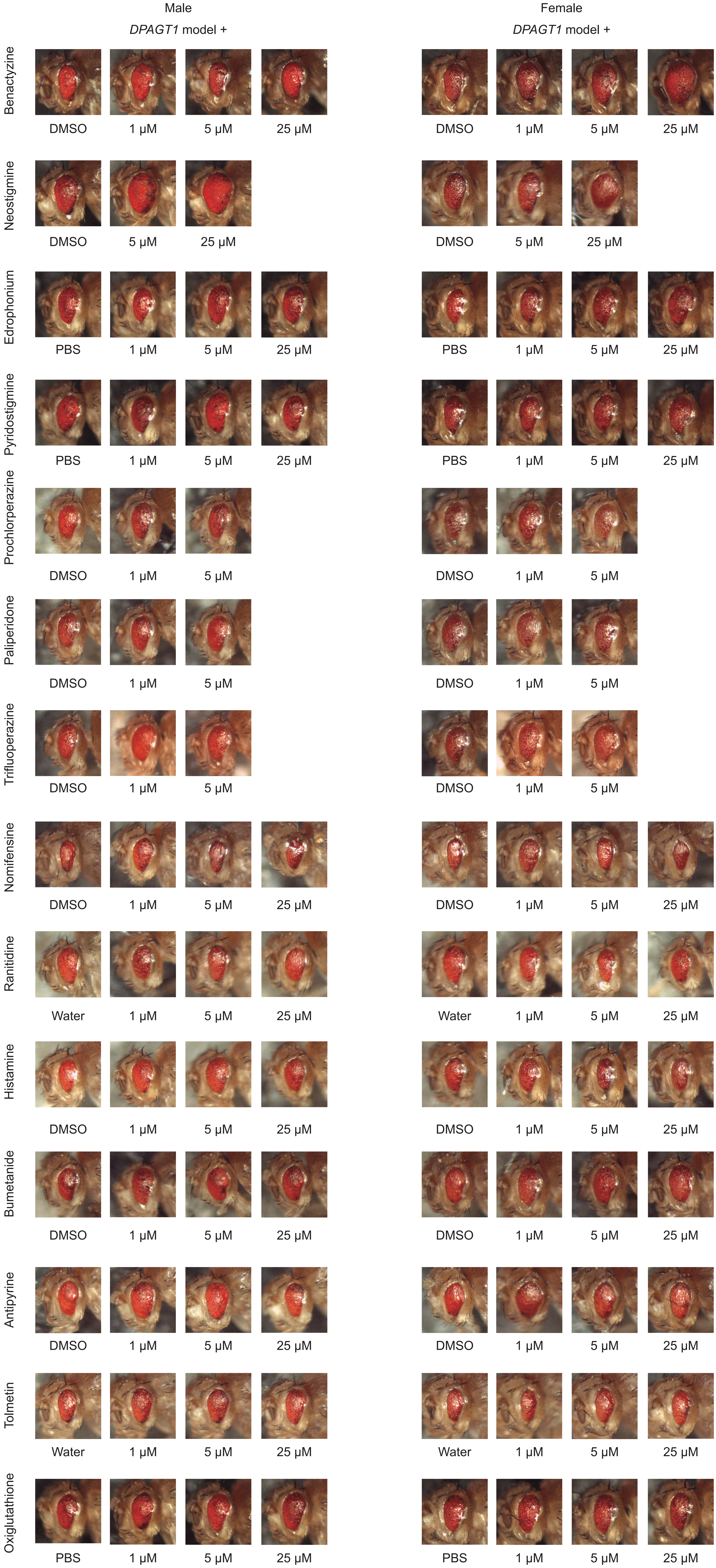
Male
DPAGT1 model +
Female
DPAGT1 model +
Benactyzine
DMSO
1 μM
5 μM
25 μM
Neostigmine
DMSO
5 μM
25 μM
Edrophonium
PBS
1 μM
5 μM
25 μM
Pyridostigmine
PBS
1 μM
5 μM
25 μM
Prochlorperazine
DMSO
1 μM
5 μM
Paliperidone
DMSO
1 μM
5 μM
Trifluoperazine
DMSO
1 μM
5 μM
Nomifensine
DMSO
1 μM
5 μM
25 μM
Ranitidine
Water
1 μM
5 μM
25 μM
Histamine
DMSO
1 μM
5 μM
25 μM
Bumetanide
DMSO
1 μM
5 μM
25 μM
Antipyrine
DMSO
1 μM
5 μM
25 μM
Tolmetin
Water
1 μM
5 μM
25 μM
Oxiglutathione
PBS
1 μM
5 μM
25 μM
