# Supplementary material for: A drug repurposing screen reveals dopamine signaling as a critical pathway underlying potential therapeutics for the rare disease DPAGT1-CDG
Source: PLoS Genet. 2024 Oct 28;20(10):e1011458. doi: 10.1371/journal.pgen.1011458 (PMC11542785; doi:10.1371/journal.pgen.1011458)
Supplement: S4 Fig — This includes both male and females flies and is complementary to S3 Table. (PDF) [file pgen.1011458.s008.pdf]

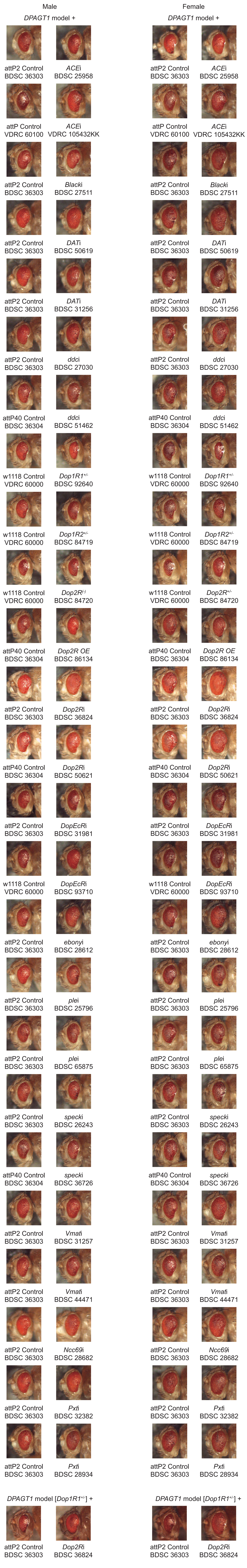
Male
DPAGT1 model +
attP2 Control
BDSC 36303
ACEi
BDSC 25958
attP Control
VDRC 60100
ACEi
VDRC 105432KK
attP2 Control
BDSC 36303
Blacki
BDSC 27511
attP2 Control
BDSC 36303
DATi
BDSC 50619
attP2 Control
BDSC 36303
DATi
BDSC 31256
attP2 Control
BDSC 36303
ddci
BDSC 27030
attP40 Control
BDSC 36304
ddci
BDSC 51462
w1118 Control
VDRC 60000
Dop1R1+/-
BDSC 92640
w1118 Control
VDRC 60000
Dop1R2+/-
BDSC 84719
w1118 Control
VDRC 60000
Dop2R(-)
BDSC 84720
attP40 Control
BDSC 36304
Dop2R OE
BDSC 86134
attP2 Control
BDSC 36303
Dop2Ri
BDSC 36824
attP40 Control
BDSC 36304
Dop2Ri
BDSC 50621
attP2 Control
BDSC 36303
DopEcRi
BDSC 31981
w1118 Control
VDRC 60000
DopEcRi
BDSC 93710
attP2 Control
BDSC 36303
ebonyi
BDSC 28612
attP2 Control
BDSC 36303
plei
BDSC 25796
attP2 Control
BDSC 36303
plei
BDSC 65875
attP2 Control
BDSC 36303
specki
BDSC 26243
attP40 Control
BDSC 36304
specki
BDSC 36726
attP2 Control
BDSC 36303
Vmati
BDSC 31257
attP2 Control
BDSC 36303
Vmati
BDSC 44471
attP2 Control
BDSC 36303
Ncc69i
BDSC 28682
attP2 Control
BDSC 36303
Pxfi
BDSC 32382
attP2 Control
BDSC 36303
Pxfi
BDSC 28934
DPAGT1 model [Dop1R1+/-] +
attP2 Control
BDSC 36303
Dop2Ri
BDSC 36824
Female
DPAGT1 model +
attP2 Control
BDSC 36303
ACEi
BDSC 25958
attP Control
VDRC 60100
ACEi
VDRC 105432KK
attP2 Control
BDSC 36303
Blacki
BDSC 27511
attP2 Control
BDSC 36303
DATi
BDSC 50619
attP2 Control
BDSC 36303
DATi
BDSC 31256
attP2 Control
BDSC 36303
ddci
BDSC 27030
attP40 Control
BDSC 36304
ddci
BDSC 51462
w1118 Control
VDRC 60000
Dop1R1+/-
BDSC 92640
w1118 Control
VDRC 60000
Dop1R2+/-
BDSC 84719
w1118 Control
VDRC 60000
Dop2R+/-
BDSC 84720
attP40 Control
BDSC 36304
Dop2R OE
BDSC 86134
attP2 Control
BDSC 36303
Dop2Ri
BDSC 36824
attP40 Control
BDSC 36304
Dop2Ri
BDSC 50621
attP2 Control
BDSC 36303
DopEcRi
BDSC 31981
w1118 Control
VDRC 60000
DopEcRi
BDSC 93710
attP2 Control
BDSC 36303
ebonyi
BDSC 28612
attP2 Control
BDSC 36303
plei
BDSC 25796
attP2 Control
BDSC 36303
plei
BDSC 65875
attP2 Control
BDSC 36303
specki
BDSC 26243
attP40 Control
BDSC 36304
specki
BDSC 36726
attP2 Control
BDSC 36303
Vmati
BDSC 31257
attP2 Control
BDSC 36303
Vmati
BDSC 44471
attP2 Control
BDSC 36303
Ncc69i
BDSC 28682
attP2 Control
BDSC 36303
Pxfi
BDSC 32382
attP2 Control
BDSC 36303
Pxfi
BDSC 28934
DPAGT1 model [Dop1R1+/-] +
attP2 Control
BDSC 36303
Dop2Ri
BDSC 36824
